# The reprogramming impact of SMAC-mimetic on glioblastoma stem cells and the immune tumor microenvironment evolution

**DOI:** 10.1186/s13046-025-03452-1

**Published:** 2025-07-04

**Authors:** Qiong Wu, Jianan Chen, Anders E. Berglund, Dongliang Du, Robert J. Macaulay, Arnold B. Etame

**Affiliations:** 1 Department of Neuro-Oncology, H. Lee Moffitt Cancer Center & Research Institute, Tampa, FL 33612 USA; 2 Department of Quantitative Health Sciences, Division of Computational Biology, Mayo Clinic, Jacksonville, FL 32224 USA; 3 Departments of Biostatistics and Bioinformatics, H. Lee Moffitt Cancer Center & Research Institute, Tampa, FL 33612 USA; 4 Departments of Anatomic Pathology, H. Lee Moffitt Cancer Center & Research Institute, Tampa, FL 33612 USA

## Abstract

**Background:**

Intrinsically resistant glioma stem cells (GSCs) in the setting of a highly immunosuppressive tumor microenvironment (TME) remain the most predominant phenomenon leading to unfavorable therapeutic outcomes in glioblastoma (GBM). Hence there is an unmet need for novel anti-GBM therapeutic paradigms that can effectively target GSCs while simultaneously reprogramming the TME.

**Methods:**

In this study, we leverage evidence from SMAC mimetic screening to evaluate and characterize the anti-tumor and immune TME modulating impacts of the lead SMAC mimetic Xevinapant at the single cell level in GBM. We utilized viability assays and orthotopic human and murine GBM models to assess the survival impacts of Xevinapant on GSCs in vitro and in vivo. Moreover, we employed single-cell RNA sequencing (scRNA-seq) to investigate the modulation impact of Xevinapant on GBM TME. Lastly, we investigated drug combination synergies to address potential mechanisms of tolerance or resistance to Xevinapant.

**Results:**

According to our observations, in vitro exposure to Xevinapant induced apoptosis along with significant viability reduction in a dose-dependent manner, in both human and mouse GSCs. Moreover, Xevinapant treatment produced robust anti-tumor effects in vivo and significantly prolonged animal overall survival. Based on single-cell RNA seq analysis, Xevinapant did not only enhance GSCs apoptosis but also activated antitumor effector immune response leading to favorable reprogramming of immunosuppressive TME. Furthermore, we established and queried Xevinapant therapeutic signatures to the LINCS database in an effort to identify small molecules that could reverse treatment-induced tolerance to Xevinapant. We have identified a novel set of candidate small molecules with robust synergy when combined with Xevinapant.

**Conclusions:**

In summary, Xevinapant exhibits robust anti-tumor activity on GSCs and favorable immune modulation of the TME in GBM, hence providing a rationale for further clinical investigation in GBM.

**Supplementary Information:**

The online version contains supplementary material available at 10.1186/s13046-025-03452-1.

**Table:** 

Text box 1. Contributions to the literature
• Glioblastoma remains one of the deadliest cancers with limited effective treatments, partly due to therapy-resistant stem-like cells and an immunosuppressive tumor environment. This study advances the field by demonstrating how a targeted drug, Xevinapant, can address both issues simultaneously
• This study adds novel insights into how reprogramming the tumor immune landscape can enhance treatment impact.
• The use of single-cell analysis provides high-resolution evidence for drug effects often missed in traditional studies.
• These findings support new therapeutic strategies that combine targeted and immune-based treatments in hard-to-treat cancers.

## Introduction

Glioblastoma (GBM) is the most common malignant primary brain tumor. It is highly resistant, brain-invasive, and uniformly fatal with a median survival of 14 months despite current aggressive multimodal therapeutic efforts [1, 2]. Novel impactful therapies are desperately needed. Major contributory factors to therapeutic futility include intra-tumoral molecular heterogeneity [3–6], persistence of stem-like cancer cells [7–12], and a highly heterogeneous and immunosuppressive tumor microenvironment (TME) [13–15]. A pivotal advance in understanding GBM pathogenesis was the identification of a subpopulation of tumor-initiating cells with stem-like properties. This subpopulation is commonly referred to as glioma stem cells or GBM stem cells (GSCs). These cells exhibit self-renewal, multilineage differentiation, and the ability to recapitulate tumors in vivo, setting them apart from the bulk tumor population [12]. Subsequent studies demonstrated that GSCs are not only more tumorigenic but also more resistant to conventional treatments such as radiation and chemotherapy, underscoring their role in tumor recurrence [16]. These insights have provided strong justification for developing therapies that specifically target the GSCs population, aiming to achieve more durable treatment responses and prevent relapse. GBM TME components include tumor cells, immune cells, glial cells, and other cell types that collectively facilitate tumor progression and immunosuppression [14, 17]. Dynamic interactions between the GBM tumor and non-tumor cellular populations are critical for tumor propagation, brain infiltration, immune evasion, and therapeutic resistance [14, 15]. Well-recognized mechanisms of immune evasion in GBM can occur via (i) an immunosuppressive TME enriched with regulatory T cells (Tregs), myeloid-derived suppressor cells (MDSCs), and tumor-associated macrophages (TAMs); (ii) activation of suppressive immune checkpoint molecules such as PD-L1 [13, 18, 19]. The above mechanisms facilitate immune surveillance evasion, tumor invasion, and tumor progression [20, 21]. Further, immune suppression can facilitate tumor survival adaptation through the acquisition of genetic mutations and epigenetic modifications [20, 21]. Hence, there is a strong rationale to develop anti-GBM therapeutic paradigms that can effectively target both GBM immune evasion as well as GSCs.

Second Mitochondria-derived Activator of Caspases (SMAC) mimetics are a class of small molecules designed to mimic the function of SMAC, a natural protein that targets inhibitor of apoptosis proteins (IAPs) by neutralizing the inhibitory effects of IAPs on caspases [22, 23]. IAPs are characterized by the presence of baculoviral IAP repeat (BIR) domains for interactions with caspase [24–26], and play a critical role in cell death [27], immunity [28], and inflammation [28]. SMAC mimetic molecules mimic the AVPI sequence which refers to the N-terminal tetrapeptide (Ala-Val-Pro-Ile) of SMAC, also known as DIABLO. This Ala-Val-Pro-Ile sequence is critical for SMAC’s pro-apoptotic function of SMAC, and bind to the BIR3 domain of IAP proteins which frees up caspases to promote apoptosis in cancer cells [29]. Moreover, recent studies have also begun to explore their impact on the TME and emerging evidence suggests that SMAC mimetics can promote anti-tumor immunity through multiple mechanisms including activation of dendritic cells (DCs) [30]; activation of microglia [31]; regulation of tumor-associated macrophages [32]; and T cell infiltration [30]. For instance, SMAC mimetic treatment has been shown to modulate microglial activation states and reshape the immune landscape in GBM, suggesting immunomodulatory effects beyond tumor-intrinsic cytotoxicity [31]. Additionally, 3D tumor models that better mimic the GBM microenvironment have revealed complex effects of SMAC mimetics on both tumor and stromal compartments [30]. Given the above mechanisms, SMAC mimetics can synergize with checkpoint inhibitors [33, 34].

We screened several SMAC mimetics with a focus on discovering candidates that target both GSCs and GBM immune evasion. Xevinapant emerged as the most effective SMAC mimetic for GSCs. Xevinapant is a potent and orally bioavailable SMAC mimetic, that targets IAP family member cIAP1 (gene *BIRC2*), cIAP2 (gene *BIRC3*) and XIAP [35]. Notably, previous work has implicated *BIRC3*: i) in GBM therapeutic resistance [36]; (ii) as a biomarker for the mesenchymal GBM habitat in patients [37]; (iii) as a mediator of hypoxia-driven survival adaptation through HIF1α [37]; and (iv) as a facilitator of stemness reprogramming in GBM [38]. Predictably, pharmacologic targeting of *BIRC2* and *BIRC3* will not only induce GBM tumor apoptosis but should also impact the TME as a whole. So far, Xevinapant is being investigated in several clinical trials [39–41].

In this study, we sought to further investigate and characterize the anti-tumor and immune TME-modulating impacts of Xevinapant in human and murine GBM models at the single-cell level, offering a high-resolution view of immune remodeling and resistance-associated phenotypes in vivo. We also evaluated associated mechanisms of therapeutic resistance and therapeutic synergy to Xevinapant in GBM. Our study, which represents the first comprehensive characterization of the impact of Xevinapant on the GBM TME repertoire demonstrates robust anti-tumor activity and favorable immune modulation of the TME, all highly supportive for further clinical investigation of Xevinapant in GBM.

## Methods and materials

### Cell culture and reagents

The patient-derived GSCs were well characterized and cultured in NS-A medium (90% NeuroCult NS-A Basal Medium Human plus 10% Human NeuroCult NS-A proliferation Supplements (StemCell Technologies, Vancouver, Canada) in 3D sphere. The *Trp53*^*+/−*^ and *Nf1*^*+/−*^ mouse GSC was cultured in NS-A medium (90% NeuroCult NS-A Basal Medium Mouse/Rat plus 10% NeuroCult NS-A proliferation Supplements (StemCell Technologies). Complete medium was supplied with recombinant human EGF and FGF (R&D System, MN, USA) and 100 units/mL penicillin-100 µg/mL streptomycin (Life Technologies, CA, USA). Anti-cIAP1 (mouse and human), anti-cIAP2 (human) and anti-XIAP (mouse and human) antibodies were obtained from Cell Signaling Technology (MA, USA). Anti-cIAP2 (mouse) antibody was obtained from R&D systems (MN, USA). Cleaved Caspase-3 antibody for IHC was purchased from Abcam (Cambridge, United Kingdom). Xevinapant and ST-059620 (7,8,3’,4’-Tetrahydroxyflavone) were obtained from MedChemExpress (NJ, USA).

## Cell viability

5000 cells were seeded in 96-well plate. DMSO or Xevinapant with indicated dosing were added the next day in triplicates and treated for 72 h. Following drug treatment, cell viability was assessed using CellTiter-Glo 2.0 Cell Viability Kit (Promega, WI, USA). Luminescence was measured by BioTek HT plate reader. Data analysis and calculation were carried out using GraphPad Prism 9 (MA, USA).

## Development of xevinapant resistant GSCs and drug-response curve

To establish the Xevinapant resistant cells, GSC2 and GSC4 were exposed to stepwise increasing concentrations of Xevinapant (up to 250 µM) over a period of 75 days. In short, GSCs were initially cultured in the presence of 15.6 µM Xevinapant. The concentration was then increased by 2-fold for 15 days (approx. every three passages) until it reached 250 µM. For the drug combination, GSCs were culture with Xevinapant as well as 25nM ST-059620. Viable cell numbers were counted every 5 days (every passage), and then were seeded with 1:10 ratio. The percentile of seeded cells to initial cell number (day 0) was recorded. For drug-response curve, 5,000 cells/well were plated in 96-well plates were treated 96 h later with various doses of Xevinapant and cell viability was measured using CellTiter-Glo 2.0.

## Caspase 3/7 activity

5000 cells were seeded in 96-well plate. DMSO or Xevinapant with indicated dosing were added the next day in triplicates and treated for 72 h. We then utilized Caspase-Glo 3/7 Assay System (Promega, WI, USA) to quantify caspase-3/7 activity in cells that are undergoing treatment. Upon caspase cleavage, distinct fluorophores are released, Ex/Em = 535/620 nm for Caspase 3/7, which can be readily monitored according to the manufacturer’s instructions.

## Western-blot assay

50–100 µg of heat-denatured proteins were loaded on 4–15% precast polyacrylamide gel (Bio-Rad, CA, USA). The proteins were then transferred to PVDF membranes (Bio-Rad), which were blocked with 5% non-fat milk solutions for 1 h at room temperature. The target proteins were then detected by the primary antibody at 4 °C overnight, washed with 0.1% Tween-TBS and incubated with appropriate secondary antibody for 1 h at room temperature. The membranes were then washed, and the target proteins were detected with luminol reagent and X-ray film (Santa Cruz Biotechnology, TX, USA).

### Animals

6–8 weeks female NCRNU athymic mice and C57BL/6 mice were purchased from Taconic Biosciences.

## Orthotopic GSC xenograft model by intracranial injection

For orthotopic model, xenograft tumors were established by intracranially injecting 2–3 × 10^5^ indicated GSCs in a 3–4 µL volume of PBS in the right striatum of NCRNU athymic mice on a Stoelting Digital Stereotaxic Instrument (Stoelting, IL, USA). After implantation, the mice were randomized into distinct groups for treatment with Xevinapant (50 mg/kg/day) or the control vehicle by oral gavage for 2 weeks or until the humane endpoint. For survival studies, animals were followed every day until they lost 20% of body weight or had trouble ambulating, feeding or grooming.

## Mouse GSC flank model and immunohistochemistry

1 × 10^6^ mGSC were injected in the flank of female C57BL/6 mice (6–8 weeks). Two weeks later, mice were subjected to vehicle or Xevinapant treatment (50 mg/kg, oral, daily). Tumor samples were harvested on day 30 and were fixed with 10% neutral-formalin buffer for 24 h. The samples were then dehydrated, paraffin-embedded and sectioned. Sections were dewaxed, treated with 3% H_2_O_2_ for 10 min and incubated with anti-cleaved Caspase 3 antibody (1:100 dilutions) overnight at 4 °C. Biotinylated secondary antibody (1:200 dilutions) was added at room temperature for 1 h, followed by the incubation with ABC-peroxidase for an additional 1 h. After washing with Tris-buffer, the sections were incubated with DAB (3, 30 diaminobenzidine, 30 mg dissolved in 100 ml Tris-buffer containing 0.03% H_2_O_2_) for 5 min, rinsed in water and counterstained with hematoxylin.

### Mouse orthotopic GSC model by intracranial injection

For orthotopic model, 1–2 × 10^4^ mGSC were intracranially injected in a 2 µL volume of PBS in the right striatum of C57BL/6 mice on a Stoelting Digital Stereotaxic Instrument (Stoelting). After implantation, the mice were randomized into distinct groups for treatment with Xevinapant (50 mg/kg/day) or the control vehicle by oral gavage. For survival studies, animals were treated for 2 weeks and followed every day until they lost 20% of body weight or had trouble ambulating, feeding or grooming. For scRNA-seq animals were treated for 7 days.

### Real-time PCR

Total mRNA was extracted from developed resistant cells using RNeasy mini-prep kit (Qiagen, MD, USA). The RNA was qualified and quantified with Nanodrop 2000 (Thermo Scientific, MA, USA). cDNA was synthesized using iScript cDNA Synthesis kit (Bio-Rad). And then Real-time PCR was performed using synthesized cDNA by iQ SYBR green Supermix buffer system (Bio-Rad) and the Bio-Rad CFX96 Touch Real-Time PCR Detection platform. The primers used in the study are listed in Supplemental Table 1.

### Data acquisition and characteristics analysis

The association between *BIRC2*/*BIRC3* expression and tumor purity and specific immune cell (macrophages, DCs, T cells, etc.) infiltration were analyzed, using TIMER 2.0 (http://timer.cistrome.org/). The results were visualized in scatter plots. RNA sequencing data and corresponding clinical information were obtained from The Cancer Genome Atlas (TCGA) database (https://portal.gdc.cancer.gov/), and the Chinese Glioma Genome Atlas (CGGA) database (http://www.cgga.org.cn/). To investigate the correlation between *BIRC2*/*BIRC3* expression and immune cell infiltration, we applied the xCell algorithm [42] and the MCP-counter algorithm [43]. These analyses were performed using the “xCell” and “MCPcounter” R packages. Additionally, we employed the GSEA (Gene Set Enrichment Analysis) method to explore the relationship between *BIRC2*/*BIRC3* expression and the infiltration of 28 immune cell types, using the “GSVA” package [44]. Gene Ontology (GO) enrichment and Kyoto Encyclopedia of Genes and Genomes (KEGG) pathway analyses were performed on the related genes. The “clusterProfiler” package in R was used to annotate and visualize GO terms and KEGG pathways [45].

### Single-cell RNA sequencing and analysis

Single-cell RNA sequencing (scRNA-seq) was performed at Molecular Genomics Core of Moffitt Cancer Center. Sequencing reads were mapped against mm10 mouse transcriptome and processed for UMI counting using Cell Ranger (v3.0, 10X Genomics, CA, USA). Clusters were identified using the by Louvain clustering implemented in FindClusters function at resolution = 0.8. Uniform manifold approximation and projection (UMAP) was used to visualize gene expression and clusters. Differential expression analysis for each cluster was performed using FindAllMarkers function in Seurat with default settings. Clusters were further annotated by comparing differential genes with canonical markers. More detailed procedures were described in Supplemental Methods.

### Analysis of the LINCS drug data

The Library of Integrated Network-based Cellular Signatures (SigCom LINCS; https://maayanlab.cloud/sigcom-lincs/) catalogs transcriptional responses following treatments with small molecules, thus representing a resource for investigating drugs in the drug tolerance network, which includes 33,621 small molecules, 248 cell lines and 1,113,059 signatures in total. We queried from the LINSC database with our signature consisting of 24 most significant up-regulated genes and 18 most significant down-regulated genes. The top signature results for the input were displayed separately by dataset, indicating reverser signatures and mimicker signatures.

### Calculation of drug-response synergy index

To quantify drug interactions between Xevinapant and ST-059620 and also to classify the interactions, we calculated combination index and isobologram. A cross-design was made to test the synergy and sensitivity of a drug pair in GSCs. We simulated isobologram for the pair of Xevinapant and ST-059620 with eight effective doses cross combinations 1/2 titrated for 72 h. Loewe additivity model was used for two drugs combination. All drug responses were determined by viability test. For synergy score measurement, data matrix was input and analyzed by SynergyFinder v3.0 (https://synergyfinder.fimm.fi).

### Statistics

Student’s t-test (for 2 condition experiments) and ANOVA (for multiple condition experiments) were employed. Survival was assessed using the Kaplan-Meier analysis method with statistical comparisons made by log rank (Mantel-Cox) comparison. Bar graphs are presented as mean ± SEM as indicated in the figure legends. Unless otherwise noted, each experiment was repeated at least three times. All the replicates and animal numbers are listed as “n” in all figure legends. All statistical tests were considered significant at *p* < 0.05. Prism 9 software (GraphPad Software) was used to create graphs and conduct statistical analyses.

## Results

### Xevinapant induces human and mouse GSC apoptosis and suppress tumor growth

**Fig. 1:**
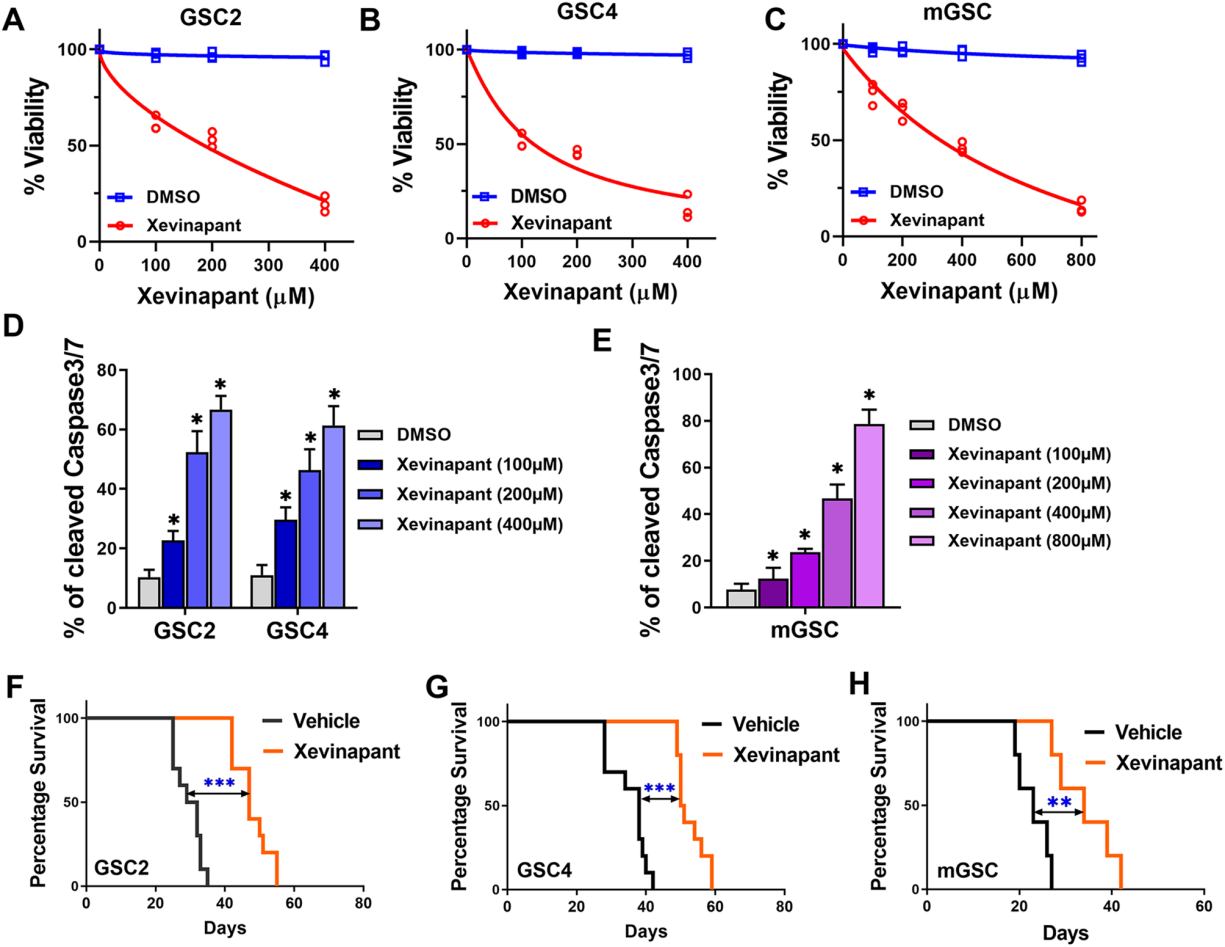
Xevinapant induces human and mouse GSCs apoptosis and suppresses tumor growth. **A**-**B**. Cell viabilities were measured following different doses of Xevinapant in human GSC2 and GSC4. **C**. Cell viabilities were measured following different doses of Xevinapant in mGSC. **D**. Caspase 3/7 activity measurement following different dosing of Xevinapant in human GSCs. *n* = 3; Mean ± SEM; *, *p* < 0.05. **E**. Caspase 3/7 activity measurement following different dosing of Xevinapant in mGSC. *n* = 3; Mean ± SEM; *, *p* < 0.05. **F**-**G**. Kaplan–Meier survival curves of immunocompromised mice bearing GSC2 and GSC4 treated with Vehicle or Xevinapant. The p-values were calculated by Mantel-Cox log-rank test. *n* = 10; **, *p* < 0.01 ***, *p* < 0.001. **H. **Kaplan–Meier survival curves of immunocompetent mice bearing mGSC treated with Vehicle or Xevinapant. The p-values were calculated by Mantel-Cox log-rank test. *n* = 5; *, *p* < 0.05

We first assessed the treatment effects of multiple SMAC mimetics on patient-derived GBM stem cells (GSC). We observed that Xevinapant was the best SMAC mimetic that suppressed GSC viability the most (Fig. S1A). We then examined Xevinapant inhibition efficiency of its molecular targets by assessing cIAP1 (*BIRC2*), cIAP2 (*BIRC3*) and XIAP protein expressions in a dose-dependent manner in GSCs (GSC2 and GSC4). The western blot result indicated that Xevinapant significantly suppressed cIAP1 expression in both GSCs at 100 μm dose, while a higher dose of 400 µM was needed for treatment for cIAP2 depletion (Fig. S1B). However, even at 400 µM Xevinapant could only suppress XIAP expression in GSC4 but not GSC2 (Fig. S1B). We next examined GSCs sensitivity to Xevinapant using cell viability assessment, and the results revealed that Xevinapant significantly suppressed GSCs cell viability in a dose dependent manner (Fig. 1A-B). Similar results were observed in GBM cell lines, including U251 and T98G (Fig. S2A-B). Meanwhile, we also assessed the impact of Xevinapant on mouse GSC. We took advantage of *Trp53*^*+/−*^ and *Nf1*^*+/−*^ mouse GSC (mGSC), which is a mesenchymal-like, highly proliferative mouse GBM stem cell line. We first treated *Trp53*^*+/−*^ and *Nf1*^*+/−*^ mGSCs with Xevinapant and examined cIAP1, cIAP2 and XIAP protein expressions in a dose-dependent manner similar to human GSCs. The western blot result indicated that Xevinapant significantly suppressed cIAP2 expression in mGSC at 100 μm dose, while totally depleting cIAP1 expression at 100 μm dose (Fig. S1C). Meanwhile, XIAP expression increased at 100 μm, 200 μm and 400 μm Xevinapant, and dropped at 800 μm dose (Fig. S1C). Next, we examined mGSC sensitivity to Xevinapant using cell viability assessment. The viability assay results revealed that Xevinapant significantly suppressed mGSC cell viability in a dose dependent manner similar to human GSCs (Fig. 1C). Similar observations were noted with other mouse GBM cell lines, including GL261 and CT-2 A (Fig. S2C-D). Using the same dose-dependent treatment scheme, we evaluated apoptosis evolution by measuring cleaved caspase 3/7 levels in both human and mouse GSCs. The results indicated that Xevinapant induced both human and mouse GSCs apoptosis also in a dose-dependent manner (Fig. 1D-E), which is consistent with the results of cell viability assessment. To further investigate the in vivo therapeutic effects of Xevinapant, we took advantage of orthotopic xenografts by intracranial implantation of GSC2 and GSC4. The animals were treated with either vehicle control or Xevinapant, respectively, daily for 7 consecutive days. The results showed that Xevinapant treatment significantly prolonged survival in animals bearing either GSC2 (*n* = 10; *p* < 0.001) or GSC4 (*n* = 10; *p* < 0.001) tumors (Fig. 1F-G). Thus, Xevinapant significantly induces human GSCs apoptosis, suppresses tumor growth, and further improves survival. Next, we utilized both flank and orthotopic xenograft to further investigate the in vivo therapeutic effects of Xevinapant on tumor growth kinetics using mGSC model. At treatment end points, Xevinapant treated flank tumors showed significantly slower growth kinetics compared to vehicle control (Fig. S3A-B). Evaluation of harvested flank tumors by immunohistochemistry (IHC) staining revealed robust cleaved caspase 3 tissue expression in response to Xevinapant treatment, indicating that there was significant activation of apoptosis in GBM xenografts, compared to the control group (Fig. S3C). In the orthotopic xenograft model, the animals harboring mGSC tumors were treated with vehicle control and Xevinapant, respectively. Mice harboring mGSC treated with Xevinapant exhibited significantly increased animal survival (*n* = 5; *p* < 0.01; Fig. 1H). These results collectively are highly supportive of the premise that Xevinapant can prevent apoptosis evasion and improve GBM outcomes.

### BIRC2 and BIRC3 expressions are associated with immune cell infiltration in GBM.

**Fig. 2:**
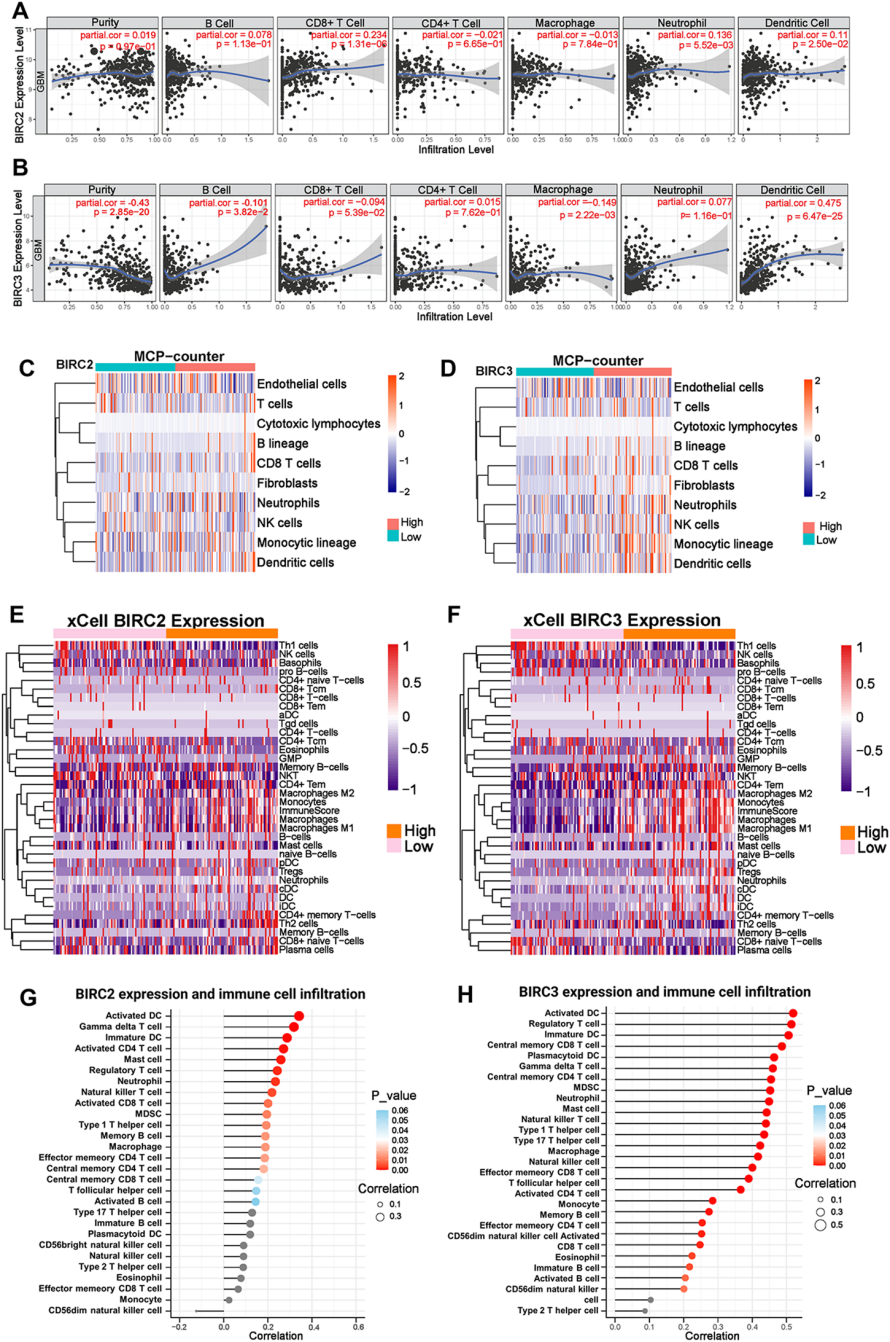
BIRC2 and BIRC3 expressions are associated with immune cell infiltration in GBM. **A**-**B**. The association between *BIRC2*/*BIRC3* expression and immune cell infiltration in GBM based on TIMER analysis. **C**-**F**. The association between *BIRC2*/*BIRC3* expression and immune cell infiltration in GBM based on MCP-counter (**C** & **D**) and xCell (**E** & **F**) analysis using TCGA database. **G-H**. Lollipop diagram displaying the correlation between *BIRC2*/*BIRC3* expression and immune cells infiltration using TCGA database

*BIRC2* (cIAP1) and *BIRC3* (cIAP2) are the members of the IAPs family that are often overexpressed and have been implicated in the pathogenesis of various malignancies including GBM [46]. Given that *BIRC2* and *BIRC3* were the downstream targets of Xevinapant, we were interested in further investigating the relationship between *BIRC2*/*BIRC3* and the TME in order to gain insights as to how Xevinapant could be leveraged to favorably modulate the GBM TME. We first evaluated for potential correlations between *BIRC2*/*BIRC3* expression and the infiltration levels of immune cells by taking advantage of TIMER database [47]. Interestingly, based on our results, *BIRC2* expression did not correlate with either tumor purity or most of the immune cell infiltration (Fig. 2A), and only showed weak negative correlation with CD8^+^ T cell infiltration (Rho = − 0.234, *p* = 1.31 × 10^− 6^, Fig. 2A**)**. Meanwhile, *BIRC3* was negatively correlated with tumor purity (Rho = − 0.43, *p* = 2.85 × 10^− 20^, Fig. 2B) and positively correlated with dendritic cell infiltration (Rho = − 0.475, *p* = 6.47 × 10^− 25^, Fig. 2B), suggesting that BIRC3 is associated with GBM TME infiltration. Next, we utilized MCPcounter and xCell algorithms to further analyze and assess the immune cell infiltrations using The Cancer Genome Atlas (TCGA) datasets (Fig. 2C-F**)**, and Chinese Glioma Genome Atlas (CGGA) datasets (Fig. S4A-D). These results suggested that *BIRC2* expression was slightly associated with CD4^+^ T cell infiltration only in the TCGA dataset (Fig. 2E), while high *BIRC3* expression was accompanied by high infiltration state of Tregs, macrophages and DCs (Fig. 2D&F, Fig. S4B&D), which are essential for antigen presentation and the activation of T cells leading to immune evasion by suppressing anti-tumor immune responses. *BIRC3* expression was highly indicative of immunosuppression within the TME, hindering effective anti-tumor responses. Furthermore, both *BIRC2* and *BIRC3* expressions were positively correlated with the infiltration of DCs, different subsets of T cells, and neutrophils based on single-sample Gene Set Enrichment Analysis (ssGSEA) algorithm (Fig. 2G-H, Fig. S4 E-F), while only *BIRC3* expression was associated with macrophage infiltration (Fig. 2H, Fig. S4 F**)**. Collectively, these results strongly supported a positive association of BIRC2/BIRC3 expression and immunosuppressive cell infiltration states in GBM. Thus, we hypothesize that the targeting *BIRC2*/*BIRC3* by Xevinapant can favorably impact the TME in GBM.

### Xevinapant treatment reprograms TME cellular distribution and activates immune response.

**Fig. 3:**
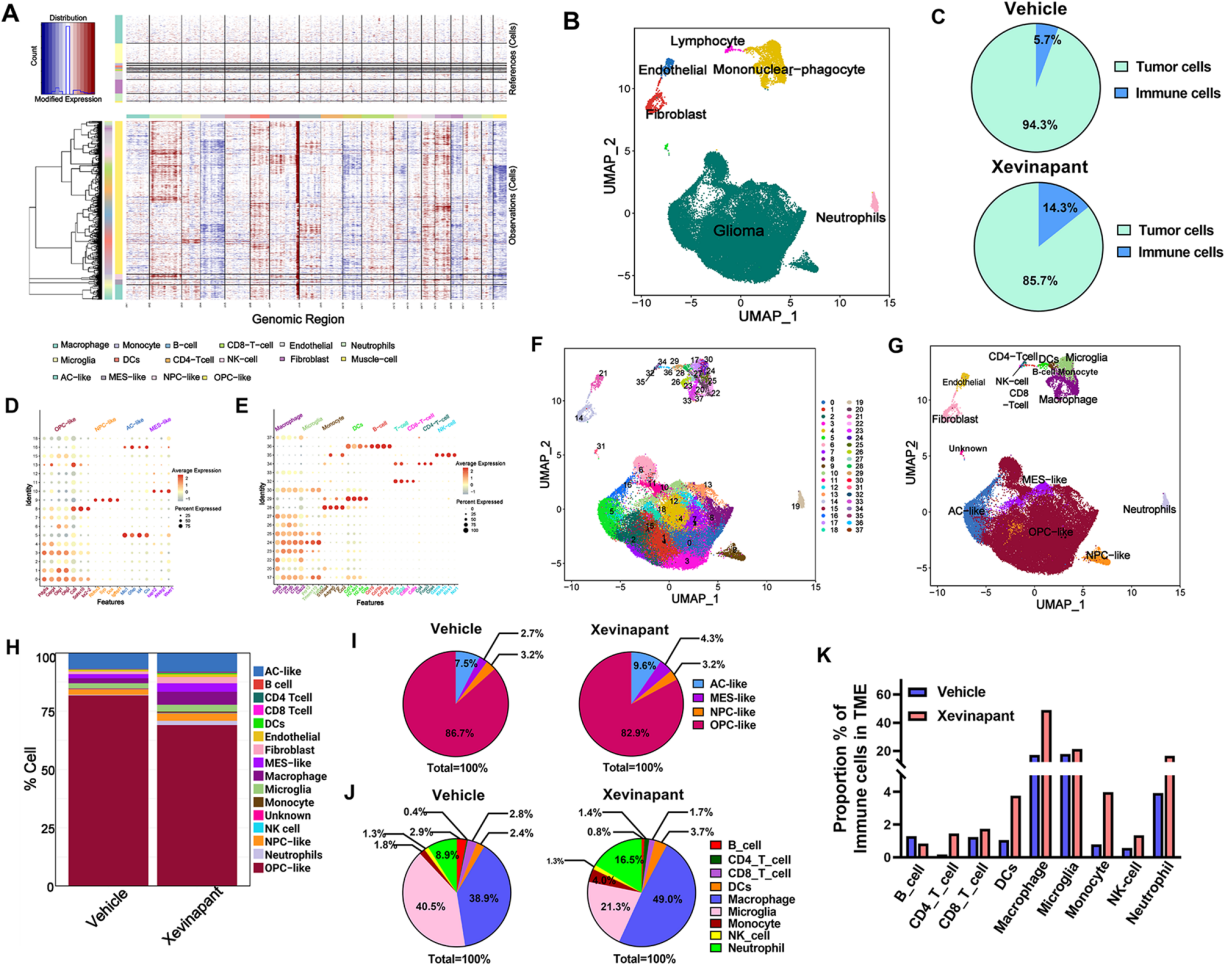
Xevinapant treatment reprograms cell distribution and activates immune response. **A** Inference of copy number variation analysis based on average expression. Each row corresponds to a cell type. **B** Different colors labeled for main cell type clusters, respectively. **C** Tumor cell and immune cell ratio in vehicle and Xevinapant tumors respectively. **D**-**E**. Dot plot displays the represented markers for each cell cluster, including MES-like (Mesenchymal-like), AC-like (Astrocyte-like), NPC-like (Neural Progenitor Cell-like), OPC-like (Oligodendrocyte Progenitor Cell-like), macrophages, microglia, monocyte, B cells, CD4 T cells, CD8 T cells, DCs, endothelial cells, fibroblast, muscle cells, neutrophils, and NK cells. **F**. Different colors labeled for specific sub-clusters, respectively. **G**. All cell types were re-clustered from and visualized in UMAP plot. Color encodes different cell clusters.** H**. Column chart demonstrates the proportions of each cell type in the tumor. **I**. Part of the whole chart demonstrates the proportions and ratios of each tumor cell type including AC-like, MES-like, NPC-like and OPC-like cells. **J**. Part of the whole chart demonstrates the proportions and rations of each type of immune cell that normalized to total immune cell ratio in their own TME. **K**. Comparison of immune cell proportion (in their own TME) in vehicle and Xevinapant treatment groups

In order to comprehensively assess the impact of Xevinapant on the cellular and molecular heterogeneity of the TME, we took advantage of single-cell RNA sequencing (scRNA-seq) before and after Xevinapant treatment. We developed *Trp53*^*+/−*^ and *Nf1*^*+/−*^ mGSC orthotopic model in C57BL/6J mice and treated them with either vehicle control or Xevinapant daily for 7 days. Cells for sequencing were isolated from these orthotopic tumors (*n* = 4 from each group) right after treatment. After QC analysis (Fig. S5 A-C), InferCNV was used for exploring this single-cell RNA-seq first to identify large-scale chromosomal copy number variations (Fig. 3A). Copy-number variation helps to distinguish the tumor cells from normal cells. After normalization of gene expression and principal component analysis (PCA), uniform manifold approximation and projection (UMAP) analyses revealed major cell types including malignant cells (GBM tumor cells); as well as non-tumor cells such as lymphocytes, endothelial cells, mononuclear-phagocyte type cells, fibroblasts, and neutrophils (Fig. 3B). Notably, we calculated tumor cells and immune cells distribution and found that Xevinapant treatment dramatically increased immune cell distribution almost 3 folds (from 5.7 to 14.3%) in the whole tumor tissue (Fig. 3C), and correspondingly, tumor cells distribution was decreased (Fig. 3C), indicating that Xevinapant induced immune response and demonstrably reprogrammed the TME within the whole tumor. We further used multiple specific markers to separate different sub-clusters in the malignant tumor cells and immune cells (Fig. 3D-E). By applying the annotation with singleR and cell type specific markers, we then merged small cell clusters into big cell lineages based on the shared transcriptomes (Fig. 3E-F). Malignant cells including MES-like cells (Mesenchymal-like, marked with *Ndrg1*, *Akap12* and *Wwtr1*), AC-like cells (Astrocyte-like, marked with *Mlc1*, *Gfap*, *Id4* and *Clu*), NPC-like cells (Neural Progenitor Cell-like, marked with *Rbfox3*, *Syp*, *Dcx* and *Lgfbpl1*) and OPC-like cells (Oligodendrocyte Progenitor Cell-like, marked with *Pdfgra*, *Olig1*, *Olig2*, *Cd9*, *Sox10*, *Cspg4* and *Nkx2-2*) were clustered together, whereas the non-tumor cells such as macrophages (marked with *Cd68*, *C1qa*, *C1qb*, *C1qc* and *Ctsz*), microglia cells (marked with *P2ry12*, *Tmem119* and *Tgfbr1*), monocyte cells (marked with *S100a4*, *Adgre5*, *Fgr* and *Ccr2*), DCs (marked with *Cd74*, *H2-Ab1*, *H2-Aa* and *Ciita*), B cells (marked with *VWF* and *PECAM1*), tumor-associated endothelial cells (marked with *Cd19*, *Cd79a*, *Cd79b* and *Fcmr*), T cells (marked with *Cd3e* and *Cd3d*), CD8 T cells (marked with *Cd8a* and *Cd8b1*), CD4 T cells (marked with *Cd4*, *Foxp3* and *Ctla4*), and NK cells (marked with *Gzma*, *Klrb1c*, *Klrk1* and *Ncr1*), were scattered (Fig. 3G).

Since the percentage of cell types and cellular states of tumor cells varied across samples, we had to take into account this heterogeneity that is highly inherent in GBM. We, therefore, pooled all samples in the same group together, and then we calculated all possible cell distributions (Fig. 3H). From the separated malignant cell distribution, we observed that AC-like (from 7.9 to 9.6%) and MES-like (from 2.7 to 4.3%) cell rations increased after Xevinapant treatment (Fig. 3I), indicating that Xevinapant did not only alter malignant tumor cells ratio in the whole tissue (Fig. 3C), but also affected tumor cell subtype distribution. We then calculated specific immune cell ratio in the TME, and the result indicated that Xevinapant treatment also altered immune cells distribution in the TME (Fig. 3J). To compare the specific immune cell population within the context of the entire TME rather than within the total immune cell pool, we normalized each type of immune cell population to the total cell number in the tissue (Vehicle: 5.6%; Xevinapant: 14.3%). For this analysis we set the immune cell population in the Xevinapant treatment group as 100%. This allowed us to directly compare both treatment groups assuming equal total cell counts, enabling dynamic observation of how individual immune cell population shift across the TME. Notably, Xevinapant treatment dramatically increased the proportions of several immune cell types compared to the vehicle group, including macrophage (from 17.1 to 49.0%), CD4 T cell (from 0.17 to 1.4%) and DCs (from 1.1 to 3.7%) and neutrophil (from 3.9 to 16.5%) ration compared to vehicle control (Fig. 3K).

To validate these observations, we further investigate the immune cell infiltrate profile in the GBM TME. Tumor samples were harvested immediately after treatment administration and analyzed by flow cytometry to compare the immune cell profile between vehicle and Xevinapant treatment (Fig. S6A). We first observed a significant increase in the total immune cell density of CD45^+^ cells within the TME in Xevinapant treatment group (Fig. S6 B). Next, analysis of CD45⁺ immune cells subpopulation in the TME demonstrated a broad increase in lymphocyte populations in response to Xevinapant treatment (Fig. S6C). Specifically, Xevinapant treatment induced a rapid increase of T cells, including CD4^+^ T cells and CD8^+^ T cells (Fig. S6D-E). Additionally, increases of myeloid cell populations were detected in the Xevinapant treatment group, including macrophage (Mφ, Fig. S6F), DCs (Fig. S6G) and neutrophil (Fig. S6H). All these results demonstrated that Xevinapant activates the immune response and strongly impacts the whole TME, which was immunosuppressed before treatment.

### Gene enrichment indicates related biological events induced by Xevinapant treatment

**Fig. 4:**
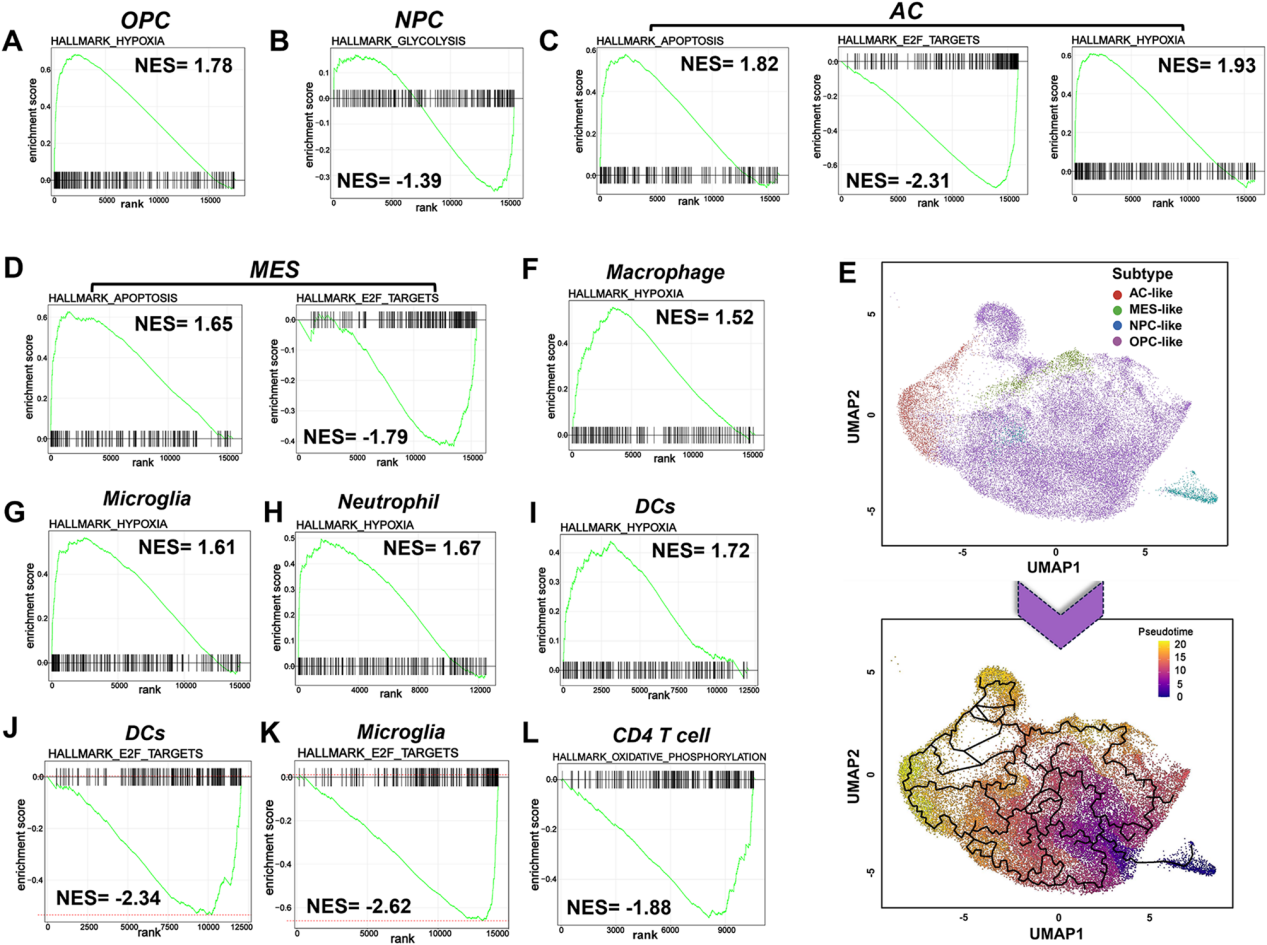
Gene enrichment indicates related biological events induced by Xevinapant treatment. **A**-**D**. Most enriched biological events in 4 kinds of subtypes of GBM tumor cell. **E**. Up: Unsupervised trajectory analysis of tumor cells was conducted using monocle. Down: Trajectory analysis using Monocle3demonstrates a major transition starting from OPC- and NPC-like to AC-like and MES-like. Each dot represents a single cell, colored by cell type in GBM. **F**-**L**. Most enriched biological events in immune cells

**Fig. 5:**
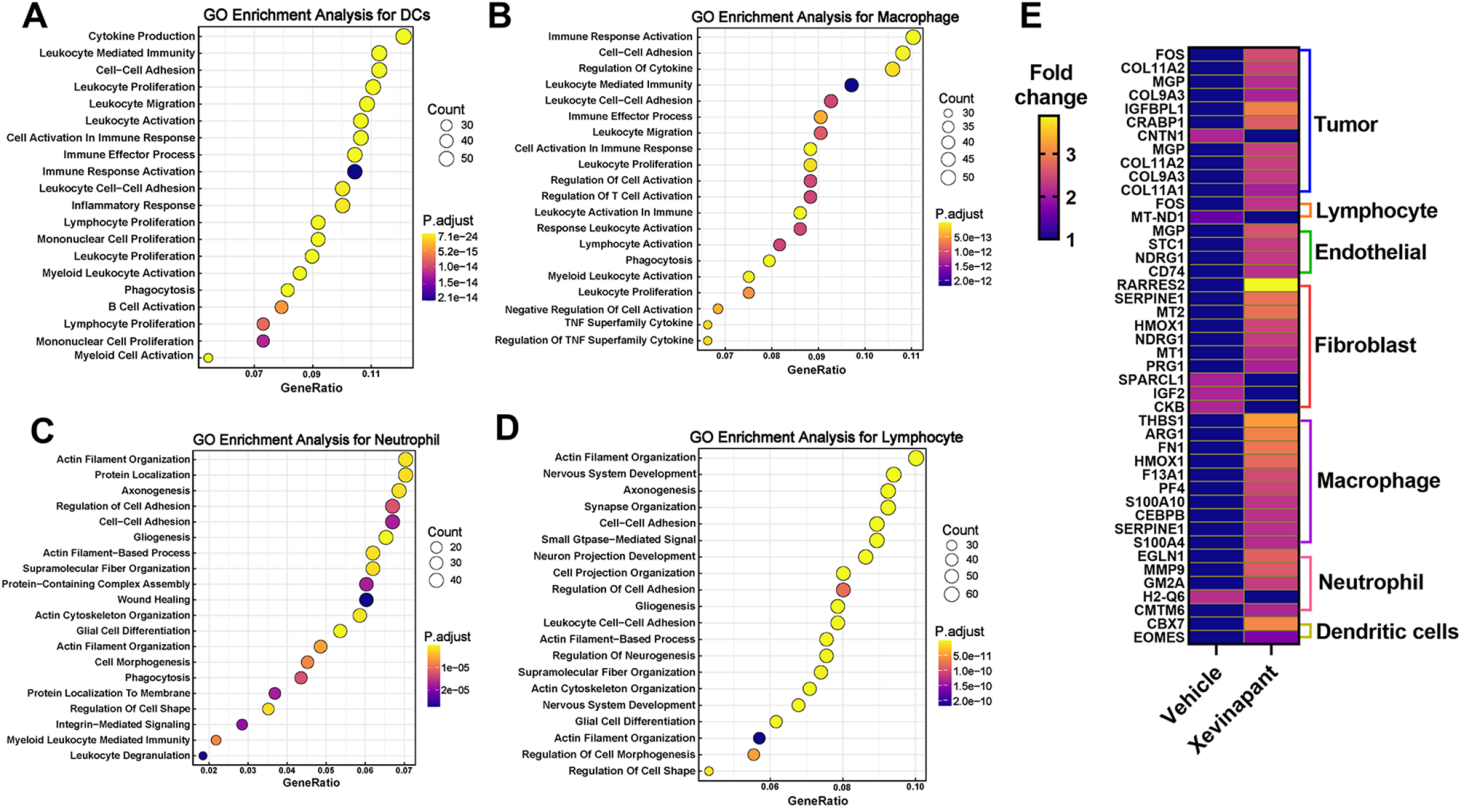
Identify the biological significance of enriched genes in different immune cells. **A**-**D**. Bubble plots showing the functional enrichment analysis of upregulated differentially expressed genes (DEGs) in Gene GO enrichment analysis of DCs (**A**), macrophage (**B**), neutrophil (**C**) and lymphocyte (**D**). **E**. The most up/down genes in different cell types. Fold change > 1

Next, we performed gene enrichment analysis to understand the impact of Xevinapant on both the malignant tumor cells and immune cells. We observed that hypoxia was the most enriched biological event in OPC-like cells while glycolysis was most enriched in NPC-like cells (Fig. 4A-B). Xevinapant mostly induced apoptosis and hypoxia in AC-like cells, while apoptosis was the most enriched biological event in MES-like cells (Fig. 4C-D). Meanwhile, Xevinapant showed strong suppression of E2F signaling in MES-like and AC-like cells (Fig. 4C-D). To explore the transition between different subtypes of GBM, we extracted all the tumor cells with different subtypes and applied unsupervised trajectory analysis using Monocle3 (Fig. 4E). Trajectory analysis revealed a major lineage starting from NPC- and OPC-like tumor cells, corresponding to the proneural (PN) type of GBM classified by TCGA, to AC-like tumor subtypes, while there was also a minor branch toward MES-like cell state after continuous Xevinapant treatment (Fig. 4E). Combining these results, we demonstrate that following Xevinapant treatment, the whole tumor became more hypoxic. Persistent OPC- and NPC-like cells became Xevinapant tolerant and evaded apoptosis subsequently transitioning towards more resistant MES- and AC-like cell types. Meanwhile, MES- and AC-like cells showed resistant properties yet both cell types still underwent apoptosis induction. To further assess the TME between vehicle and Xevinapant treatment, we analyzed gene enrichment within the different types of immune cells. Interestingly, hypoxia signaling was induced and enriched in macrophage, microglia, neutrophil and DCs after Xevinapant treatment (Fig. 4F-I), while Xevinapant suppressed E2F signaling in DCs and microglia (Fig. 4J-K). Notably, the oxidative phosphorylation signaling was inhibited in CD4 T cells (Fig. 4L), while the TME recruited more CD4 T cells after Xevinapant treatment (Fig. 3J). We then utilized Gene Ontology (GO) enrichment analysis to identify the biological significance of enriched genes in different immune cells. Interestingly, GO enrichment analysis demonstrated that, after Xevinapant treatment, cytokine production is enriched in DCs (Fig. 5A), while macrophages were correlated with immune response activation (Fig. 5B). Whereas the neutrophil and lymphocyte were undergoing actin filament organization, indicating their migration and subsequent activation of overall immune response (Fig. 5C-D). We then used gene alterations in malignant tumor cell, DCs, macrophage, lymphocyte, endothelial cells, fibroblast, and neutrophil as a signature profile for Xevinapant treatment (Fig. 5E). Notably, we observed that *MMP9* expressed was only altered in Neutrophils with Xevinapant treatment (Fig. S7A); *Arg1*, as an immunosuppression marker was altered in macrophages (Fig. S7B); *Thbs1* alteration was enriched in macrophages as well as fibroblast (Fig. S7C); *Lgfbpl1* alteration was enriched only in NPC-like cells, while *Col11a1* was enriched in most of the OPC-like cells (Fig. S7D-E). We also found that these cell type-specific signature genes are highly correlated in different ways (Fig. S8). Specifically, in malignant tumor cells, major correlation is through shared signaling pathway network (Fig. S8A); in macrophage, these genes correlate by co-expression (Fig. S8B); in endothelial cells, these genes correlate through related protein physical interaction (Fig. S8C); in fibroblast, genes correlate through co-expression and related protein co-localization (Fig. S8D); and in neutrophil, major correlation is protein physical interaction (Fig. S8E). All these observations suggest that Xevinapant treatment did not only suppress tumor progress and induce apoptosis but also reprogram the whole tumor and associated TME. Taken together, Xevinapant treatment impacts GBM tumors in three major aspects, (i) overcomes tumor apoptosis evasion (by IAPs inhibition) and attenuates tumor progression; (ii) activates immune response and disrupts immunosuppressive environment; (iii) stimulates malignant tumor cells towards treatment-resistant states following sustained exposure. All these results above from Figs. 2, 3, 4 and 5 indicated that Xevinapant treatment positively reprograms and disrupts GBM tumor immunosuppressive environment, however, adaptive resistance is inevitable.

### Identify effective drug combinations to overcome Xevinapant resistance 

**Fig. 6:**
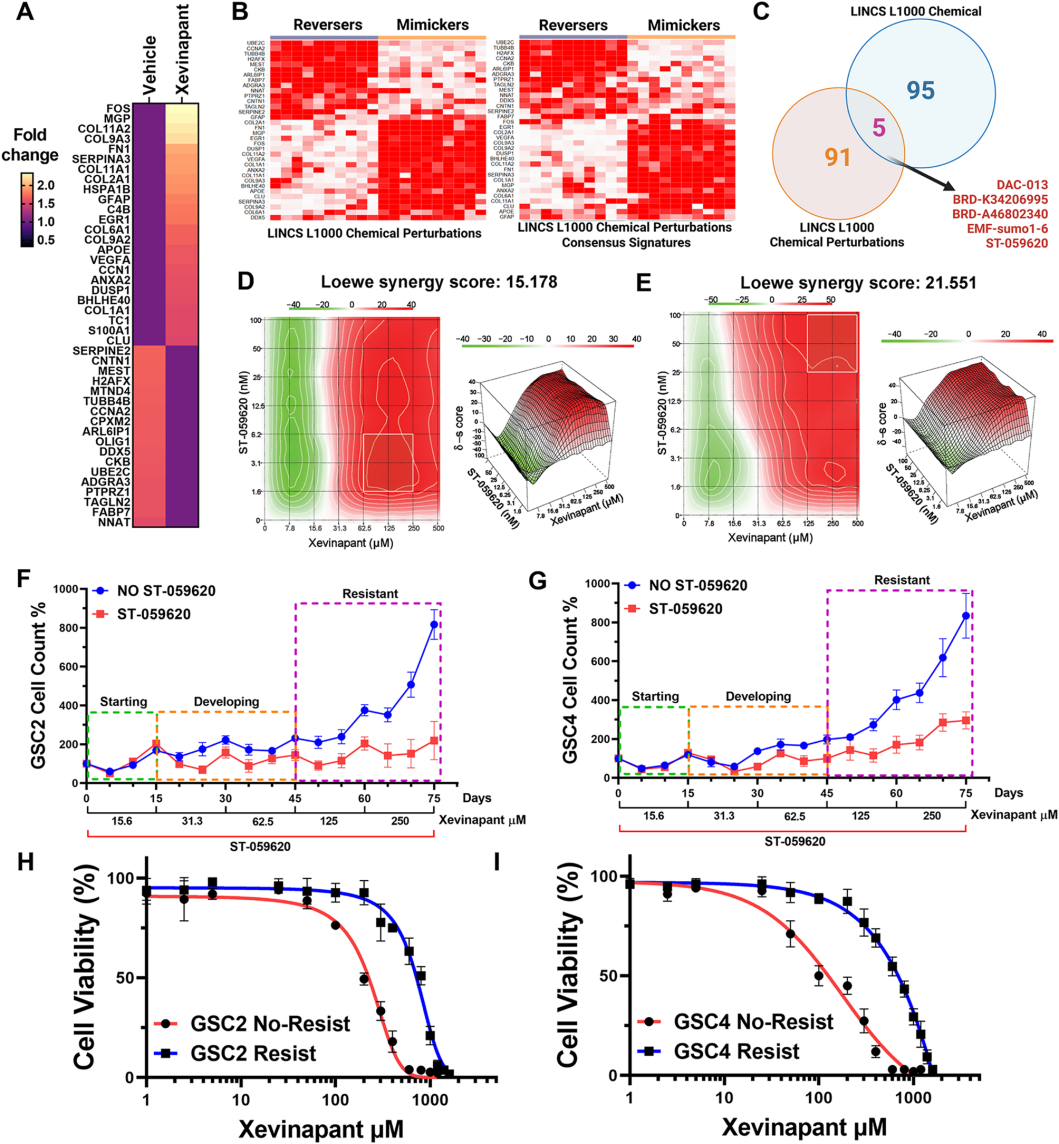
Identify effective drug combinations to overcome Xevinapant resistance. **A**. Gene signature was used for querying LINCS. **B**. The mimickers and reversers in LINCS L1000 Chemical Signature and LINCS L1000 Chemical Perturbations according to our gene signature. Clustergrams for the top 10 up- and down-regulating signatures of the input gene set were shown. **C**. Venn diagram of cross-analysis of reversers from two different libraries above. **D**-**E**. Synergy index of agents Xevinapant and ST-059620 in GSC2 (**D**) and GSC4 (**E**) were analyzed by SynergyFinder. *n* = 3. GSC2: Synergy score = 15.178. GSC4: Synergy sore = 21.551. **F**-**G**. Development of Xevinapant-resistant GSCs with or without ST-059620. *n* = 3; Mean ± SEM. H-I. Xevinapant response curves in developed acquired resistant and none-resistant GSCs. *n* = 3; Mean ± SEM

Taking all the evidence together, we found out that Xevinapant suppressed tumor growth in vivo by apoptosis induction and reprogramed immunosuppressive TME. However, Xevinapant treatment induces tumor evolution towards a resistant state with MES- and AC-like properties and highly hypoxic tumor TME. To validate this resistant phenotype in vivo, we took advantage of ex vivo drug response assay using tumor cells freshly isolated from Xevinapant-treated and vehicle control mice (Fig. S9A). After 3 days of treatment, there was no difference in viability between the vehicle and treatment groups (Fig. S9B). However, cells from treated tumors exhibited significantly reduced sensitivity to Xevinapant, in terms of viability after 6 days of treatment, compared to cells from untreated tumors (Fig. S9B), even though the treatment had been withdrawn for 3 days (Take off experiment 2: TF2, Fig. S9C). Notably, when the treatment was administered for 3 days, withdrawn for 3 days, and then reintroduced for another 3 days, the isolated cells did not exhibit significant resistance based on viability (Take off experiment 1: TF1, Fig. S9C). All these observations confirm that Xevinapant resistance can develop in vivo and a drug holiday between short-term treatments can effectively reduce resistance. Thus, we examined if Xevinapant treatment induced resistance-related signature marker gene could be targeted with small molecule inhibitor(s) (Fig. 6A). Such molecules could potentially reverse the gene expression, revert the tolerance, and inhibit the growth and progression of GBM tumor when combined with Xevinapant. We hypothesized that genetic perturbations contributing to the signature genes are potentially parts of upregulated networks that can be targeted effectively by drugs that can disrupt those networks. We identified resistance-related up (log_2_ > 0.5) /down (log_2_<-0.38) regulated genes in tumor cells from our scRNA-seq (Fig. S10A, 69 genes in total) and further performed qRT-PCR using developed Xevinapant-resistant GSCs to validate these genes expression in GSCs (Fig. S10B, fold change > 1.5). 42 validated genes were constituted as our gene signature of acquired Xevinapant resistance (Figs. 6A and 24 most up-regulated genes and 18 most down-regulated genes).

Accordingly, we then took advantage of a public drug-response signature database Library of Integrated Network-based Cellular Signatures (LINCS), which catalogs gene expression responses to small molecule inhibitors in cancer cell lines that were analyzed as described in the Methods section. The list of potential “resistant-driven” gene signatures in GBM tumor cells after Xevinapant treatment was queried to the LINCS drug response signatures. We looked for “reversers” small molecules in LINCS L1000 Chemical Perturbation Signatures and LINCS L1000 Chemical Perturbation Consensus Signatures libraries. These reverser small molecules exhibited reversed signature gene expression pattern on cancer cells compared to our signature. (Fig. 6B, Clustergram for the top 10 up- and down-regulating signatures of the input gene set in these 2 signature libraries as reversers or mimickers). We then cross-analyzed the top 100 small molecule “reversers” within two chemical libraries and found 5 compounds overlapped including ST-059620, BRD-K3406995, BRD-A46802340, DAC-013 and EMF-sumo1-6 (Fig. 6C), which potential in combination with Xevinapant may potentially reverse treatment-induced adaptive resistance and improve therapeutic outcomes. Thus, in this study, we assessed the combination effect of Xevinapant and one of the compound candidates ST-059620 (7,8,3’,4’-Tetrahydroxyflavone). First, we quantified drug interactions between Xevinapant and ST-059620 in GSCs (GSC2 & GSC4), and we calculated the drug combination index utilizing the Loewe drug interactions model. We applied eight doses for each drug. The drugs were combined on a grid, where each drug was linearly increased in one axis. All drug responses were determined by viability assessment. As shown (Fig. 6D-E), two-drug combinations demonstrated potent synergistic effects in GSCs (synergy score = 15.178 and 21.551). We also queried the list of potential “resistant” gene signatures after Xevinapant treatment to the LINCS gene expression libraries including LINCS shRNA, LINCS CRISPR knockout, LINCS CRIPSR and LINCS overexpression signatures. No overlap gene was detected but there were several common genes with three or two signature libraries overlap (Fig. S9B-C). Next, we investigated whether ST-059620 can prevent or reverse the evolution of GSCs tolerance/resistance to Xevinapant treatment. We monitored the evolution of Xevinapant resistance in GSCs (GSC2 & GSC4) during Xevinapant treatment with ST-059620 or without ST-059620 as control. In the first 2 weeks (starting period), there was no difference between the ST-059620 group and the control (Fig. 6F-G). As the concentration increased, from 25 days (developing period), the growth rate of groups with or without ST-059620 started to diverge (Fig. 6F-G). 45 days later (resistant period), there was a significant difference between the groups with or without ST-059620, as the concentration increased to 125 and 250 µM (Fig. 6F-G), suggesting that the Xevinapant treatment GSCs had become resistant, and ST-059620 was able to reverse or prevent this evolution. We then tested drug response using these newly developed resistant/non-resistant GSCs (in Fig. 6F and G), that have been treated with Xevinapant or Xevinapant plus ST-059620. The drug-response curve indicated that treatment of GSCs with Xevinapant and ST-059620 combination was effective in preventing the “resistant” cell state (Fig. 6H-I), which validated our observation during the development of Xevinapant-resistant GSCs. These observations successfully validated our query and analysis from LINCS, demonstrated that the combination therapy of Xevinapant and selected “anti-acquired resistance” molecule is an impactful strategy that can possibly address potential Xevinapant resistance.

## Discussion

Futilities with second-line therapies for GBM, as well as recent failures in GBM immunotherapy trials, underscore the desperate need for new therapeutic paradigms in GBM [48, 49], which remains one of the most aggressive and lethal cancers. The challenges in treating GBM from several factors, (i) tumor heterogeneity; (ii) blood-brain barrier limits the efficacy of many systemic therapies; (iii) therapeutic resistance; (iv) invasive and immunosuppressive TME. Since current treatment options for GBM offer limited success, ongoing research into innovative therapeutic strategies is crucial to improving the prognosis for patients with this challenging malignancy. Further, immunosuppression remains a huge obstacle to impactful immunotherapy strategies for GBM. Here, we show that strategic modulation of the TME in GBM with SMAC mimetics can enhance anti-tumor immune response in GBM, providing a novel therapeutic avenue in GBM. Here, we have systemically characterized the impact of SMAC mimetic modulation of the GBM TME highlighting alterations in cellular and molecular heterogeneity at the single cell resolution.

In the current study, we took advantage of Xevinapant, a SMAC mimetic to facilitate apoptosis in GSCs and activate an immune response in the TME through degradation of its targets *BIRC2* and *BIRC3*. We observed that Xevinapant significantly induced apoptosis in both human and mouse GSCs. Furthermore, in our orthotopic intracranial models, Xevinapant treatment significantly prolonged animal overall survival bearing human or mouse GSCs compared to vehicle control. These observations confirmed that Xevinapant is a potent drug with potential clinical applicability for GBM. To further explore this aim, we employed scRNA-seq to comprehensively assess the effect of Xevinapant on the GBM TME. Our scRNA-seq data analysis demonstrates interesting yet complex aspects of Xevinapant treatment. Most importantly, we revealed that Xevinapant treatment positively reprogramed the immunosuppressive TME in several ways: (i) increasing the immune cells infiltration into the tumor tissue; (ii) increasing CD4 T cells, macrophages, neutrophils and DCs; (iii) inhibiting E2F signaling which is a critical transcription factor that negatively regulates the maturation of DCs; and (iv) positively regulating cytokine production and activating immune response signaling pathway. Taken together, our results suggest that Xevinapant treatment significantly induces immune response activation. These observations are consistent with other studies that SMAC mimetic treatment affects intratumoral immune cell infiltration and reprogram GBM TME [31, 33]. Activating the immune response against GBM is a critical area of research due to the tumor’s immunosuppressive environment. SMAC mimetic agents like Xevinapant that disrupt the immunosuppressive signals in the GBM TME and improve immune responses will undoubtedly provide new therapeutic potential to combine with immune checkpoint inhibitors as new treatment strategy [33]. Interestingly, while Xevinapant induced apoptosis in GBM cells, treatment-induced resistant evolution of the GBM tumor cells was inevitable. Here, we have also revealed that prolonged treatment with Xevinapant over time reprogrammed GBM tumor cells towards MES- and AC-like subtypes. AC-like and MES-like are two subtype cells which are characterized by malignancy, enhanced inflammatory response, altered metabolism and stem cell properties [50, 51]. The above phenotypes as associated with treatment resistance and immune suppression rendering AC-like and MES-like difficult-to-treat tumor cell types. However, in this study, we found that although AC-like and MES-like cells are generally resistant, Xevinapant treatment successfully induced apoptosis in both AC-like and MES-like cell types. Another noteworthy observation was that hypoxia was a major biological event associated with Xevinapant treatment. While this hypoxic TME state did not compromise Xevinapant-mediated apoptosis in GSCs, sustained tumor hypoxia from long-term treatment could promote tumor metabolic and survival adaptation. Further mechanistic studies are warranted to dissect the underpinnings of Xevinapant induced tumor hypoxia.

Therapeutic tolerance is inevitable in oncology. Although Xevinapant was highly effective in inducing GSCs apoptosis and reprogramming the immunosuppressive TME, we encountered drug tolerance. We hypothesized that Xevinapant efficacy in GBM can be improved and sustained by anticipating and prospectively targeting potential genes that drive treatment resistance. We have generated signatures of tumor resistance trajectory as impacted by Xevinapant, which represent the unique patterns of response at the molecular level. Accordingly, in an effort to identify molecules that can synergize with Xevinapant, we leveraged and queried the LINCS resource, which created a comprehensive database of cellular signatures secondary to genetic and pharmacological perturbations. Using this strategy, we have uncovered a new set of agents that can ameliorate Xevinapant tolerance in GBM. Notably, one of the agents, ST-059620, demonstrated robust in vitro synergy with Xevinapant and prevented GSCs from Xevinapant-induced resistance. With this promising result, our future studies will focus on further investigating these combinatorial synergies. It is worthwhile noting SMAC memetics have established synergies with chemoradiation [52–54], and immunotherapy [33] in GBM, however, this is first report of a new class of agents that can synergize with SMAC mimetics.

The heterogeneity of GSCs remains a significant and unresolved challenge. Numerous studies have demonstrated that GSCs display substantial diversity in its functional behavior, gene expression profiles, and therapeutic responses, reflecting the extensive intratumoral heterogeneity of GBM. The isolation and definitive identification of GSCs are complicated by the reliance on surface markers such as CD133 and CD15. These markers’ expression is often inconsistent and not exclusively restricted to GSCs populations. Moreover, in vitro culture and xenotransplantation models can introduce selective pressures that influence the apparent properties of GSCs and make it difficult to mimic the native tumor environment. Recent study emphasized that GSCs heterogeneity largely arises from intrinsic tumor plasticity shaped by dynamic interactions with the TME, challenging the notion of a static, hierarchically organized stem cell pool [55]. Advances in single-cell transcriptomics, lineage tracing, and spatial profiling have further supported the concept of a fluid, plastic GSC state rather than a fixed subpopulation. The current consensus acknowledges that GSCs cannot be adequately defined by a single marker or assay, underscoring the need for integrated molecular, functional, and spatial approaches. Continued refinement of GSC models will be critical for the development of more effective, targeted therapies against the resilient and adaptable stem-like compartment of GBM.

In our study, Xevinapant has been shown to effectively kill GSCs both in vitro and in vivo; however, its treatment can also induce a shift toward more resistant cellular states. Despite its cytotoxic potential, stem-like GBM cells often exhibit resistance to Xevinapant, reflecting the intrinsic heterogeneity of the GSCs population. These cells are not uniform, while some subsets may be more susceptible to apoptosis through IAP inhibition, the others rely on alternative survival pathways. Even within the same tumor, variability in gene expression, differentiation state, or metabolic profile can influence drug sensitivity. Xevinapant induces apoptosis by antagonizing IAPs such as cIAP1 (*BIRC2*) and cIAP2 (*BIRC3*), but GSCs frequently activate compensatory pro-survival mechanisms, including NF-κB and PI3K/AKT pathways, which can mitigate the drug’s apoptotic effects. Additionally, the TME of GBM can promote GSCs survival and resistance through hypoxia, cytokine signaling, and interactions with surrounding niche cells. TME driven upregulation of anti-apoptotic proteins or suppression of death receptor signalings may significantly reduce Xevinapant’s efficacy. Importantly, even initially sensitive GSCs can develop adaptive resistance by upregulating compensatory survival proteins such as MCL-1 or survivin in response to IAP inhibition. Thus, while Xevinapant has demonstrated the ability to kill GSCs in vitro and in vivo models, resistance arises from a complex interplay of intrinsic cellular features, microenvironmental factors, and adaptive responses. These underscore the need for rational combination strategies to overcome resistance and improve therapeutic outcomes.

## Conclusion

**Fig. 7:**
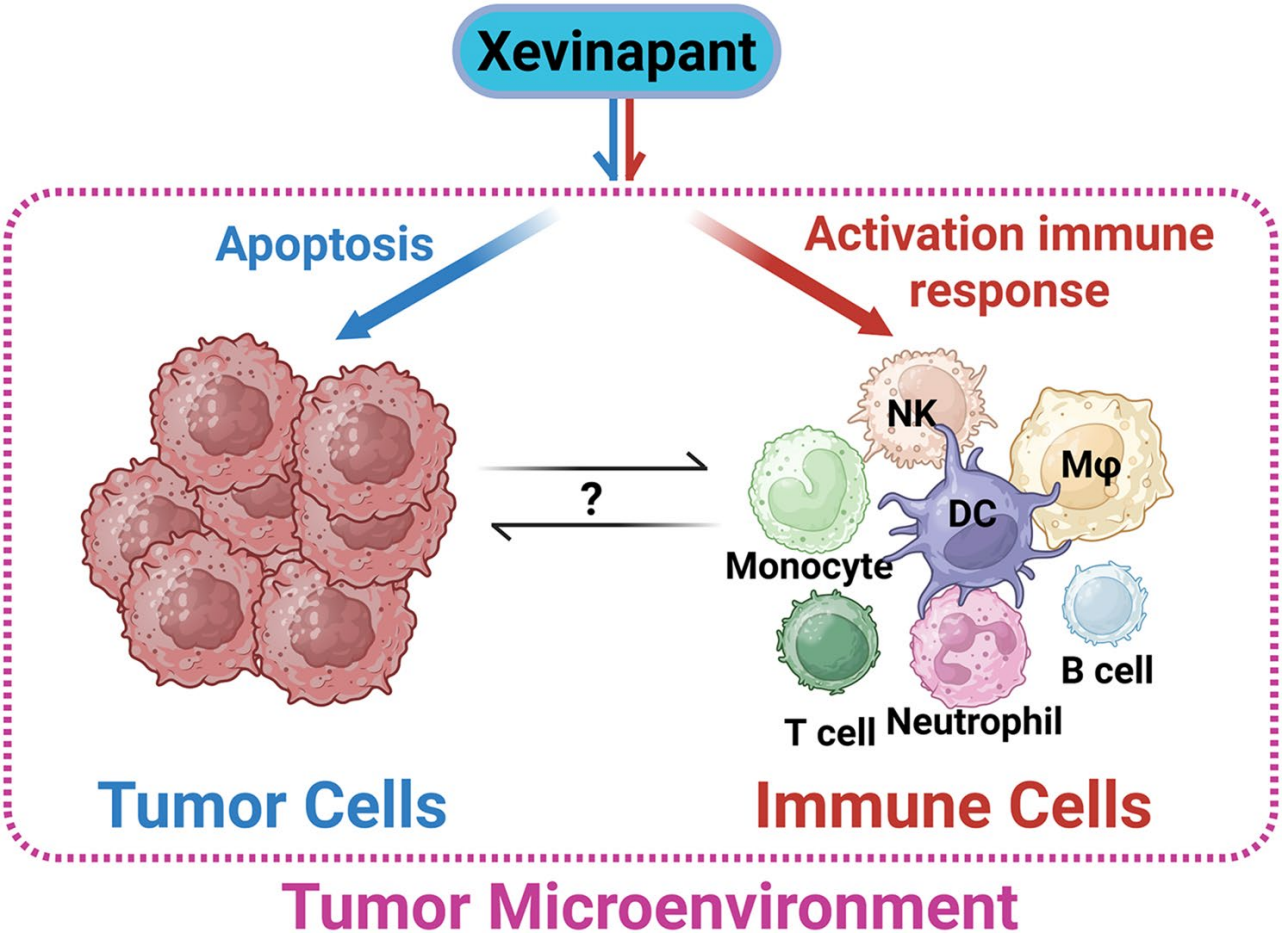
Graphical schematic summarizing Xevinapant effects on tumor cells and immune cells

In summary, we have comprehensively assessed the impact of the SMAC mimetic Xevinapant in GBM. Xevinapant appears to be a promising agent in GBM that can be leveraged not only to overcome apoptosis evasion but also to target the highly immunosuppressive GBM TME (Fig. 7). Most importantly, our work warrants further investigations into combination therapies involving SMAC mimetics and immunotherapy in GBM. Further mechanistic elucidation of synergistic mechanisms will undoubtedly provide novel therapeutic avenues for GBM patients.

## Electronic supplementary material

Below is the link to the electronic supplementary material.

Supplementary Material 1

Supplementary Material 2

Supplementary Material 3

## Data Availability

The data that supports the findings of this study are available from the corresponding author upon reasonable request. Raw scRNA-seq data generated for this study will be available in GEO (GSE296980) upon published.
