# Supplementary material for: The reprogramming impact of SMAC-mimetic on glioblastoma stem cells and the immune tumor microenvironment evolution
Source: J Exp Clin Cancer Res. 2025 Jul 4;44:191. doi: 10.1186/s13046-025-03452-1 (PMC12231904; doi:10.1186/s13046-025-03452-1)

Supplementary Table 1. **Primers used in qRT-PCR.**

Genes	F	R
FOS	TTACTACCACTCACCCGCAGACTC	GGGAATGAAGTTGGCACTGGAGAC
MGP	TCTCTCTGAACTGGCATCGTG	GAGCGTTCTCGGATCCTCTC
COL11A2	GCCTCAGCCTAGCAGAT	ATCACTCCATGGGTGTCCAATA
COL9A3	GTATTGCAGGTTCCGACGGTCT	TCTCCTTTGGGTCCGACCAG
FN1	GAGAATAAGCTGTACCATCGCAA	CGACCACATAGGAAGTCCCAG
SERPINA3	ACTCCAGACAGACGGCTTTG	ATTCTCCCATTCTCAACTCTGC
COL11A1	GTCTGTTGGTCCAGTTGGTC	TTCTCTCCTCTTTCTCCTTTTGG
COL2A1	ACCTTCATGGCGTCCAAG	AACCAGATTGAGAGCATCCG
HSPA1B	GCGAGGCGGACAAGAAGAA	GATGGGGTTACACACCTGCT
GFAP	AGAAGCTCCAGGATGAAACC	AGCGACTCAATCTTCCTCTC
C4B	GGAGGAGGAGCTGCAGTTTT	TACTCGACGTGGCCTTTGAC
EGR1	GGTCAGTGGCCTAGTGAGC	GTGCCGCTGAGTAAATGGGA
MATN1	CGGCTGACTTCAAGACCATC	CTCACAGCTTCCAGTTTCCTG
COL6A1	ACAGTGACGAGGTGGAGATCA	GATAGCGCAGTCGGTGTAGG
COL9A2	CTCGCTCTGGCGCAGATTAG	GCCCATTGTCACCGTCGAT
APOE	TGGGTCGCTTTTGGGATTAC	TTCAACTCCTTCATGGTCTCG
VEGFA	ATGACGAGGGCCTGGAGTGTG	CCTATGTGCTGGCCTTGGTGAG
SOX10	GACCAGTACCCGCACCTG	CGCTTGTCACTTTCGTTCAG
HSPA1A	AGCTGGAGCAGGTGTGTAAC	CAGCAATCTTGGAAAGGCCC
CCN1	ACTTCATGGTCCCAGTGCTC	AAATCCGGGTTTCTTTCACA
ANXA2	GAGCGGGATGCTTTGAACATT	TAGGCGAAGGCAATATCCTGT
DUSP1	GCGAAGCATCATCTCTCCCA	CACTGTTCGTGGAGTGGACA
SLC2A1	CCTGCAGTTTGGCTACAACA	GTGGACCCATGTCTGGTTG
BHLHE40	GGACAGCAAGGAGACCTACA	AGTGCTTTCACATGCTTCAAG
COL1A1	CCCCTGGAAAGAATGGAGAT	AATCCTCGAGCACCCTGA
TC1	CAAGCCATCATCATGTCCAC	GTTGCCCACGGCTTTCTTAC
S100A1	TGCTCTCACAGTGGCCTGTA	TAAGTGGGGTGAGGTGGAAG
CLU	AAACGAAGAGCGCAAGACAC	TGTTTCAGGCAGGGCTTACA
COX1	CAATGCCACCTTCATCCGAG	GATAAGGTTGGAGCGCACTG
MFAP4	CTGACAGCATGAAGGCACTC	GGGGTAGATGAGGTACACGC
H3C3	AAGACACCAATCTGTGCGCT	CGGGCAGACTTATGCCCTTT
SERPINE2	TGGTGATGAGATACGGCGTAA	GTTAGCCACTGTCACAATGTCTT
CNTN1	CAATAGTGCAGGGTGTGGAC	TGGCTAGGAGGTGCTTTCTT
KNSTRN	CCGCCTCGTTACGATGACC	TGGCCCGAGTTTGTGTGTC
MEST	ATCGGGTGATTGCCCTTGATT	GAAAGAAGGTTGATCCTGCGG
H2AFX	CGATCCTGAGTCGCCGAG	CAGCGCAGACCTTCAGGG
SELENOT	TCTCCTAGTGGCGGCGTC	GTCTATATATTGGTTGAGGGAGG
MTND1	CGGGCTACTACAACCCTTCG	AGGAGGCCTAGGTTGAGGTT
MTND4	ACAACACAATGGGGCTCACT	AATGATGTCGGGGTTGAGGG

TUBB4B	GGACAACTTCGTTTTCGGTCA	CCTTTCTCACAACATCCAGCAC
DBI	CCTAGCCCTGATTCGTTGGA	TTCTCAAACTCAGCCTCTGTCC
CCNA2	GCATGTCACCGTTCCTCCTT	GGGCATCTTCACGCTCTATTT
HNRNPK	GGCAGTGATTGGAAAAGGAG	CACTGCTGTCTGGGACTGAA
CPXM2	GTGCGCGGGAAGAAATGAC	CCTCCCTTGAGTGATGACACC
ARL6IP1	TGGTTTCCACCTGCCATCAT	CTGTTGTTCAGTGGTCCATTTAT
OLIG1	CCCCAAAAGTAGCGTAACCA	CCGGTACTCCTGCGTGTTA
DDX5	GCCGGGACCGAGGGTTTGGT	CTTGTGCTGTGCGCCTAGCCA
MTND3	CCGCGTCCCTTTCTCCATAA	GGCCAGACTTAGGGCTAGGA
CKB	CCTGCCCAGAAATGAAGC	GCACTGCCCAGGCAATAA
UBE2C	GGATTTCTGCCTTCCCTGAA	GATAGCAGGGCGTGAGGAAC
ADGRA3	CCCAGATACTCTGCCCAA	TTTCGGAGGTCCAATCTTTC
HIST1H1B	CTGCCAAACCGAAAAAGGCA	CACGCCAGCTTCCTACTTCT
PTPRZ1	GCCTGGATTGGGCTAATGGAT	CAGTGCTCCTGTATAGGACCA
TAGLN2	AGTGACATTCCCAGAGAGCC	GGCCCCTAAATTTTGGTCCC
FABP7	CCAGCTGGGAGAAGAGTTTG	CTCATAGTGGCGAACAGCAA
NNAT	GAACTGCTCATCATCGGCTG	GGAGTACCTGAACACCTCACT
GAPDH	TCAAGAAGGTGGTGAAGCAGG	TCAAAGGTGGAGGAGTGGGT

Western-blot (Fig.S1B)

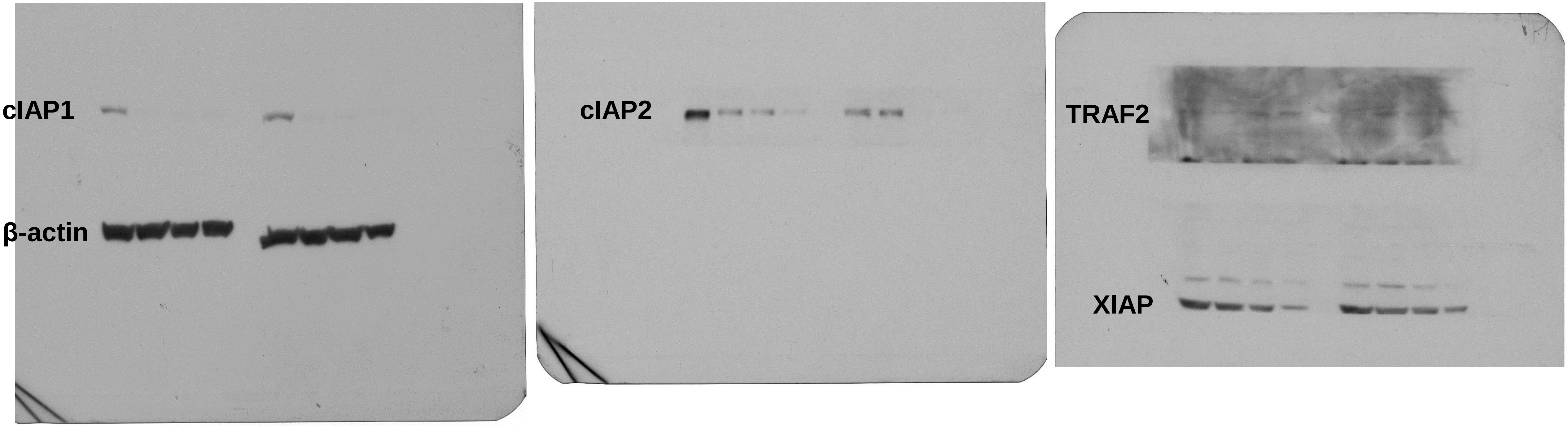

Western-blot (Fig.S1C)

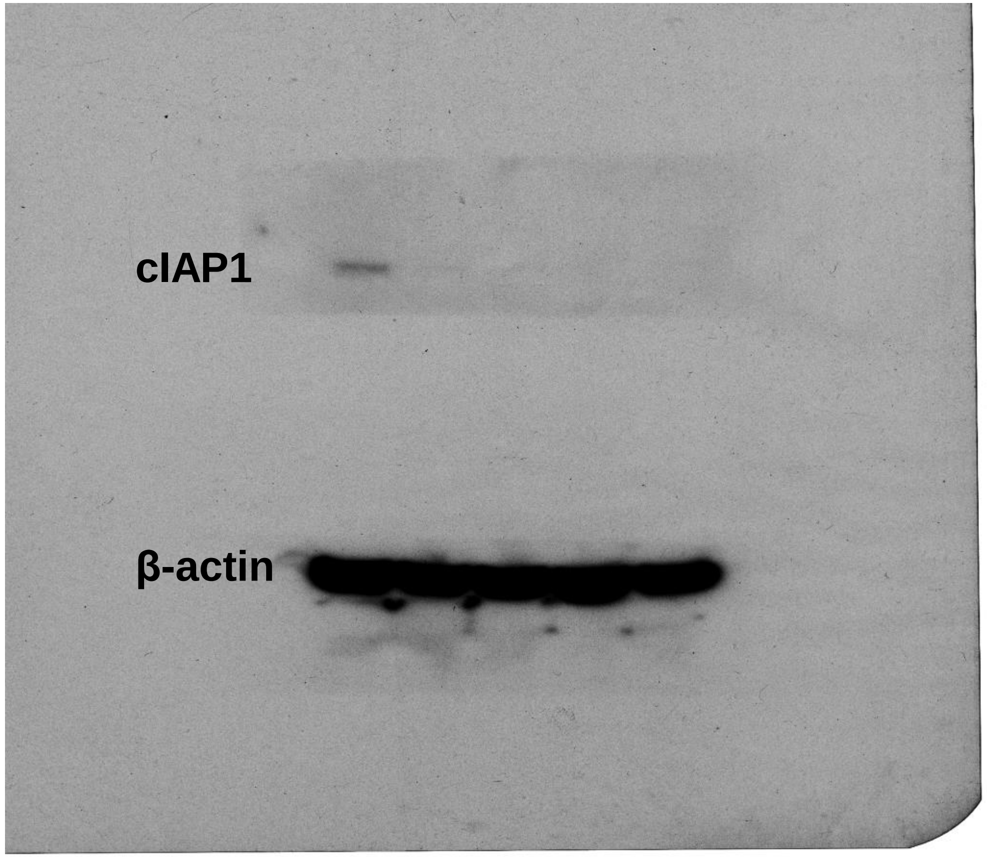

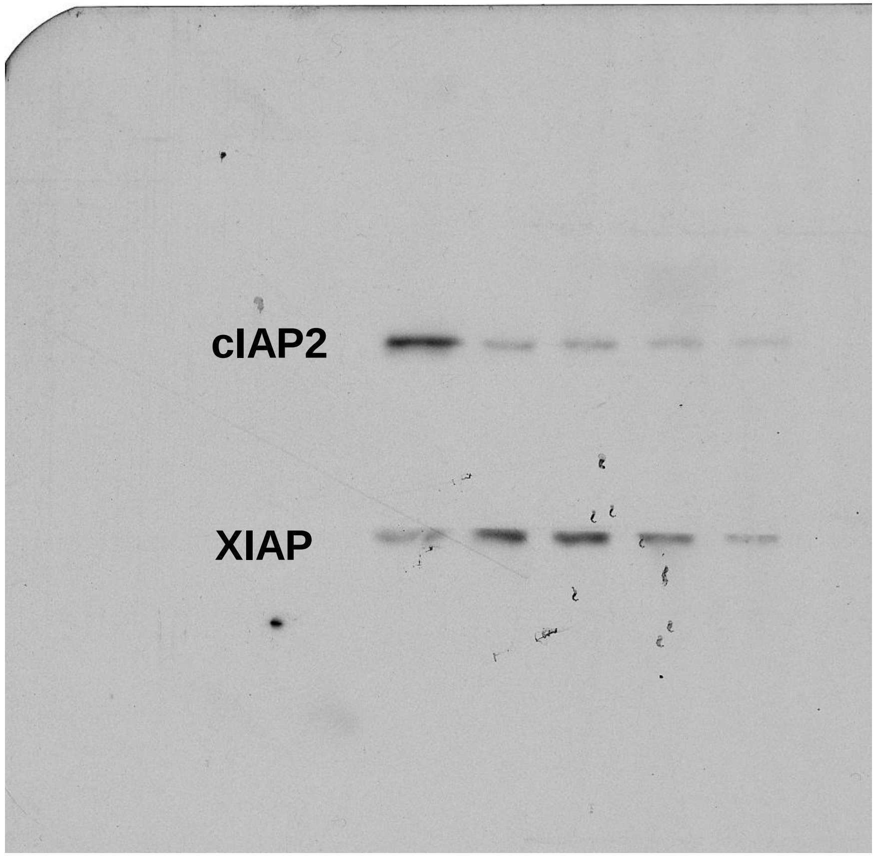

Supplement: Supplementary file 1 — Supplementary Material 1 [file 13046_2025_3452_MOESM1_ESM.pdf]
