# Supplementary material for: The reprogramming impact of SMAC-mimetic on glioblastoma stem cells and the immune tumor microenvironment evolution
Source: J Exp Clin Cancer Res. 2025 Jul 4;44:191. doi: 10.1186/s13046-025-03452-1 (PMC12231904; doi:10.1186/s13046-025-03452-1)
Supplement: Supplementary file 2 — Supplementary Material 2 [file 13046_2025_3452_MOESM2_ESM.docx]

**Supplementary Figures**

**Supplementary Figure 1**


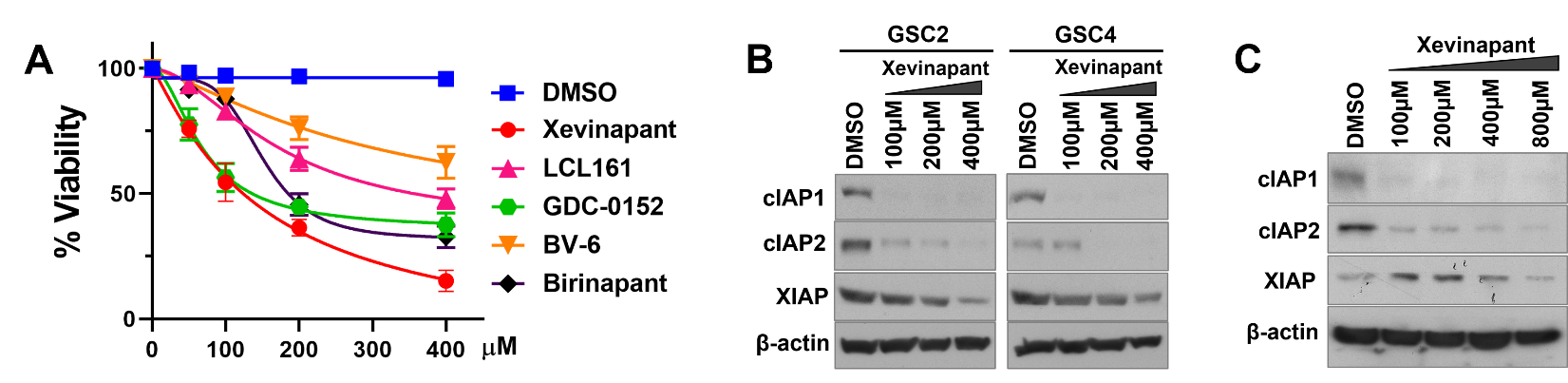


**Fig.S1** A. Cell viabilities were measured with different SMAC mimetic inhibitors. n=3, Mean ± SEM. B-C. Protein expression in human and mouse GSCs, when treated with DMSO and Xevinapant.

**Supplementary Figure 2**


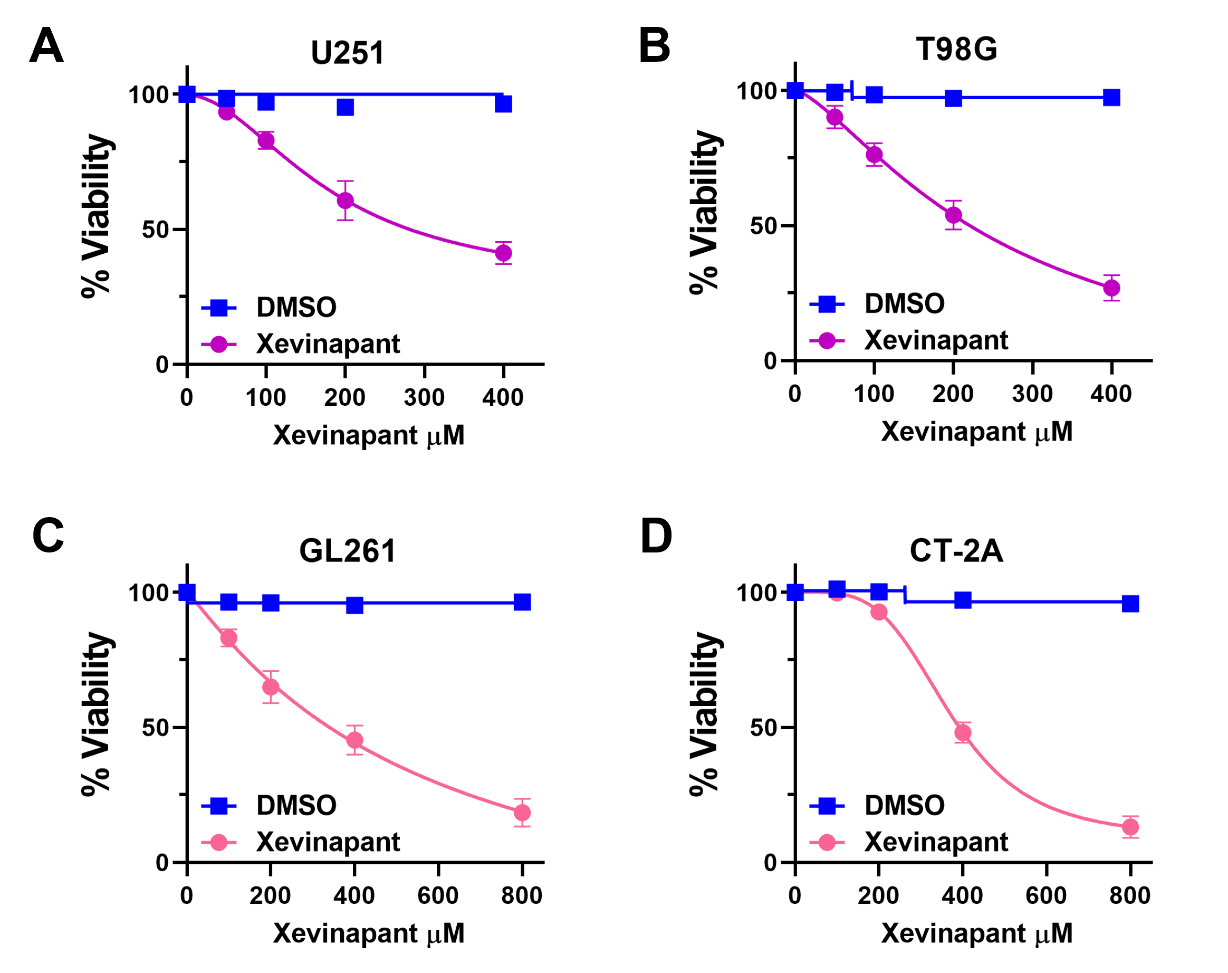


**Fig.S2** A-D. Cell viabilities were measured following different dosages of Xevinapant in human and mouse GBM cell lines. n=3, Mean ± SEM.

**Supplementary Figure 3**


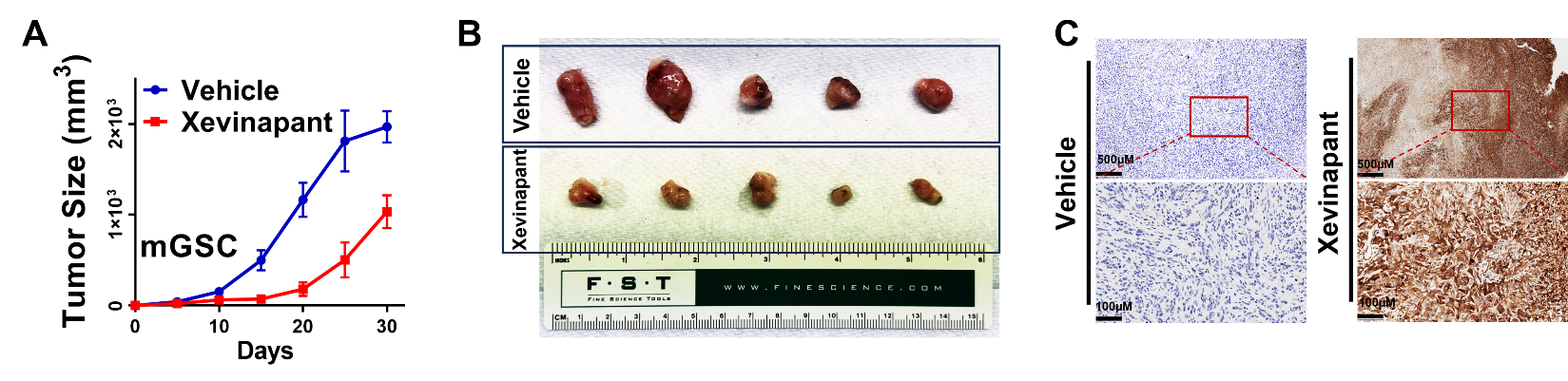


**Fig.S3** A. Tumor growth curves of mGSC, treated with vehicle or Xevinapant. n=5, Mean ± SEM. B. Mice were sacrificed on day 30, and the tumor was isolated for comparison. C. Tumor tissue was cut and fixed for cleaved caspase-3 immunohistochemistry analysis. 5 mice were included in this study and similar results were observed in each animal.

**Supplementary Figure 4**


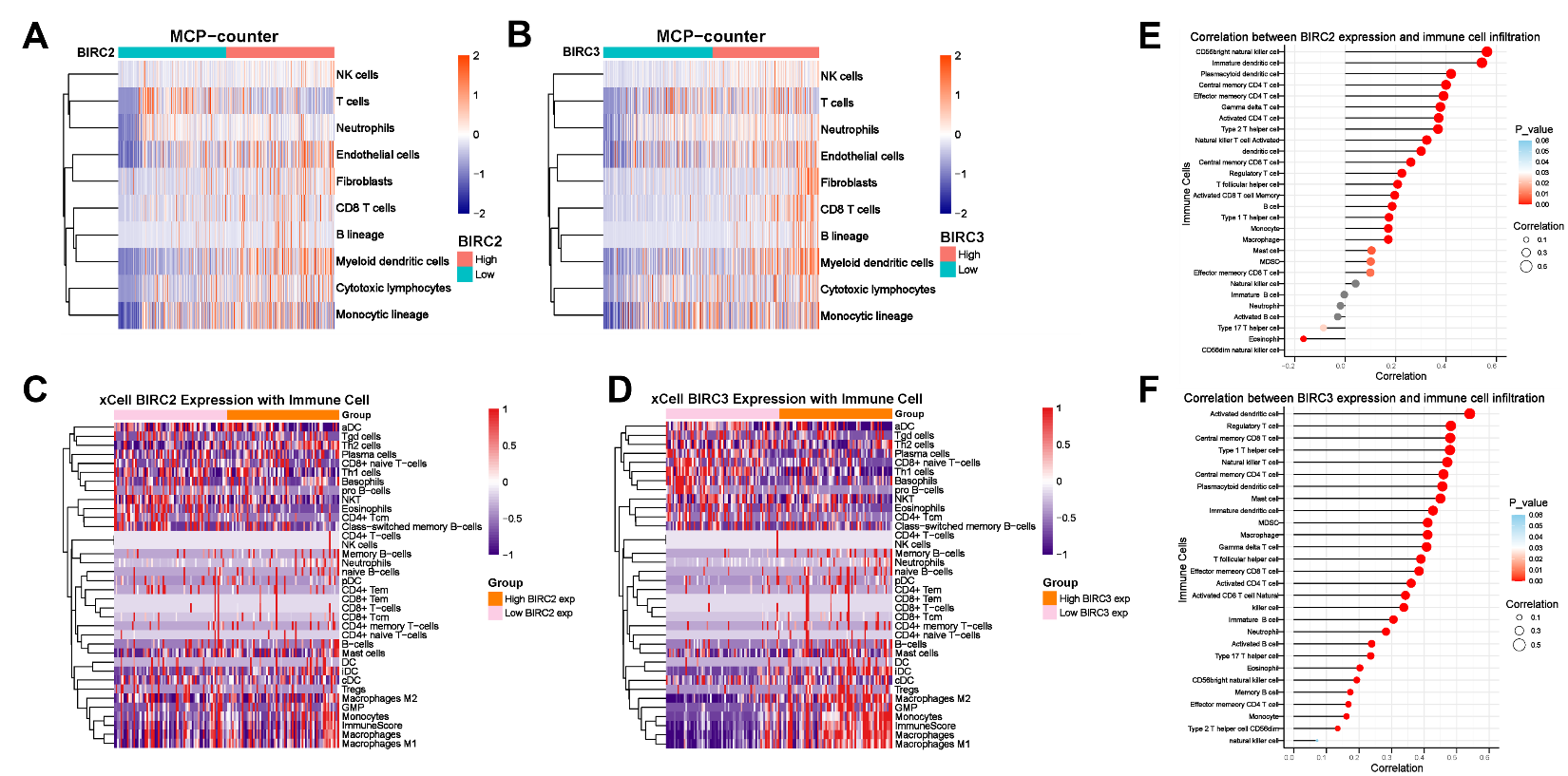


**Fig.S4** A-D. The association between *BIRC2*/*BIRC3* expression and immune cell infiltration in GBM based on MCP-counter (A-B) and xCell (C-D) analysis using CGGA database. E-F. Lollipop diagram displaying the correlation between BIRC2/BIRC3 expression and immune cells infiltration using CGGA database.

**Supplementary Figure 5**


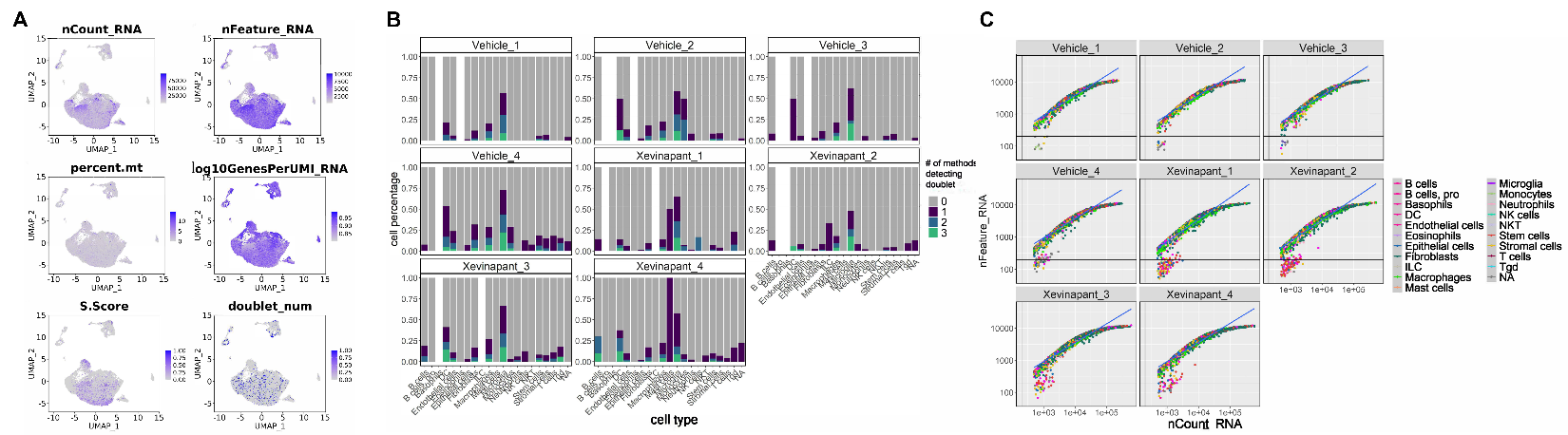


**Fig.S5** A-C. Individual sample QC in scRNA-seq.

**Supplementary Figure 6**


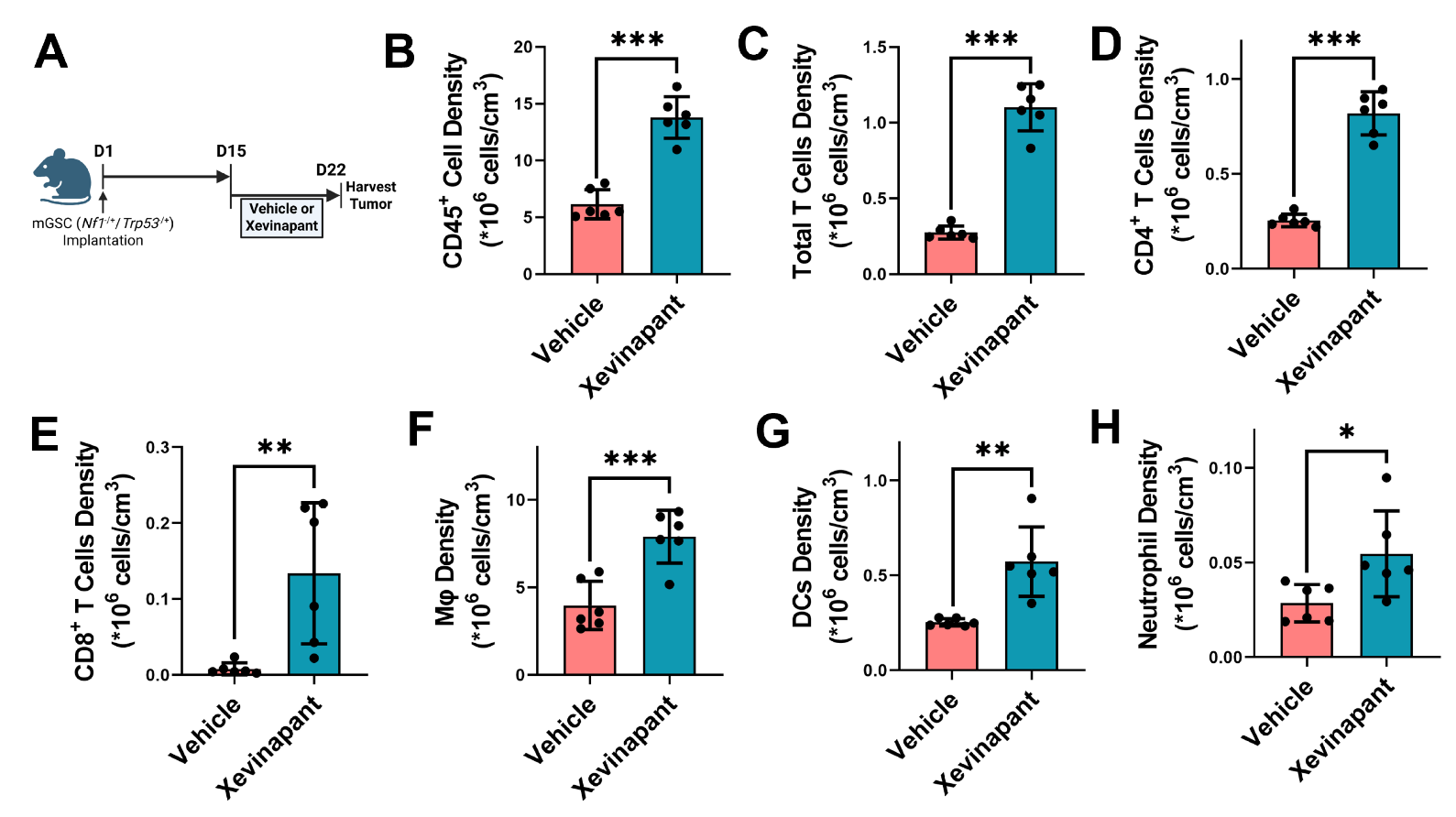


**Fig.S6** A. Timeline of the experiment. B. Total immune cells (CD45^+^) density (10^6^ cells/cm^3^) in the TME on day 22. C-E. Cell density (10^6^ cells/cm^3^) among subpopulations of T cells (CD45^+^CD3^+^CD11b^-^) in the TME. F-H. Cell density (10^6^ cells/cm^3^) among myeloid cells (CD45^+^CD3^-^CD11b^+^) in the TME including Macrophage (Mφ), DCs and Neutrophil. n=6, Mean ± SEM. *, p<0.05; **, p<0.01; ***p<0.001.

**Supplementary Figure 7**


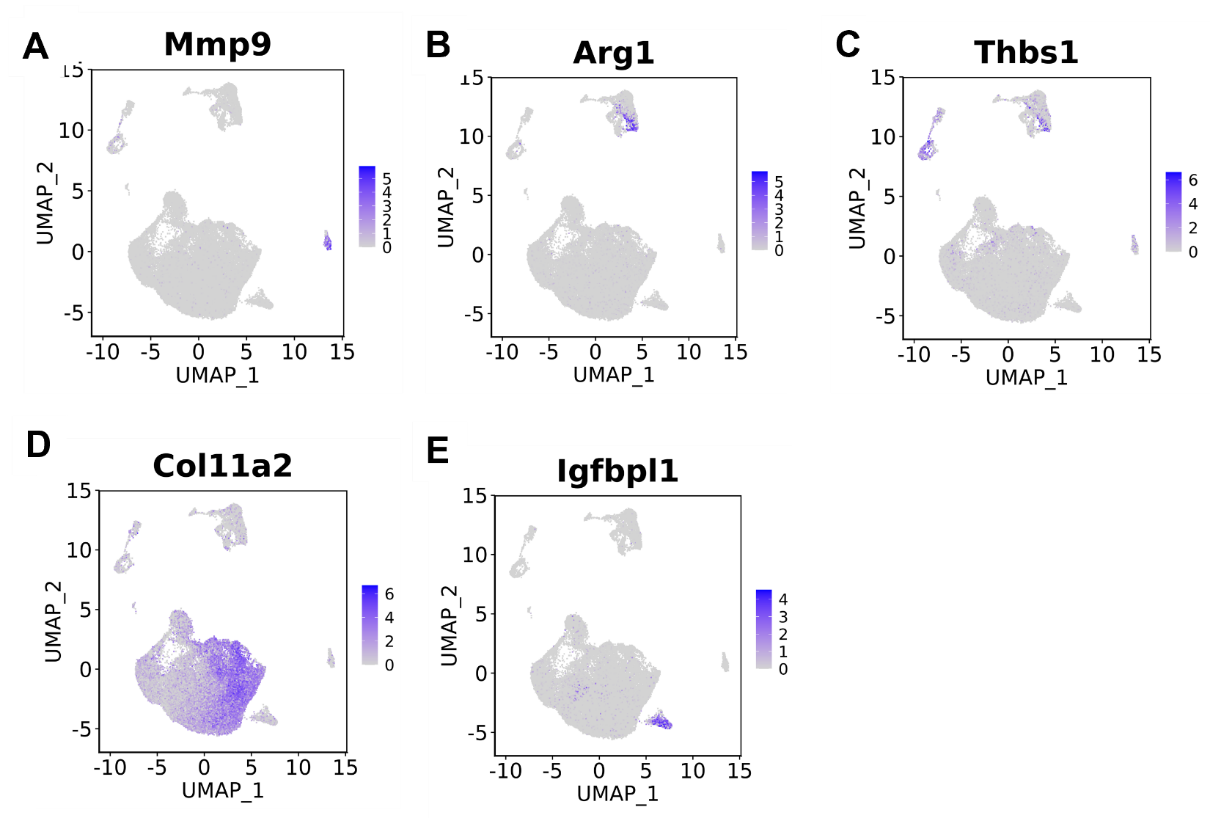


**Fig.S7** A-E. UMAP analysis of the distribution of indicated genes.

**Supplementary Figure 8**


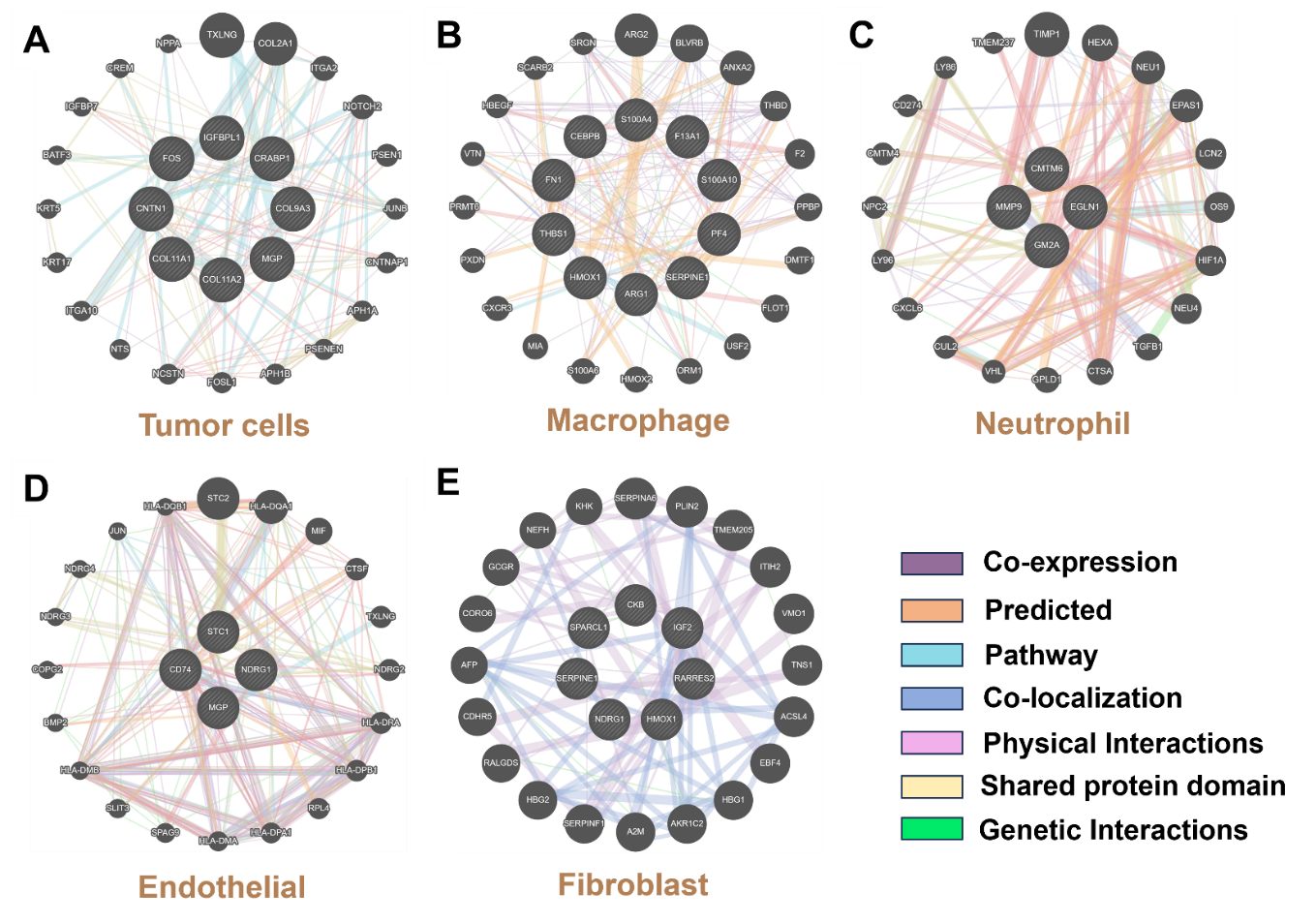


**Fig.S8** A-E. Gene correlation analyzed by GeneMANIA.

**Supplementary Figure 9**


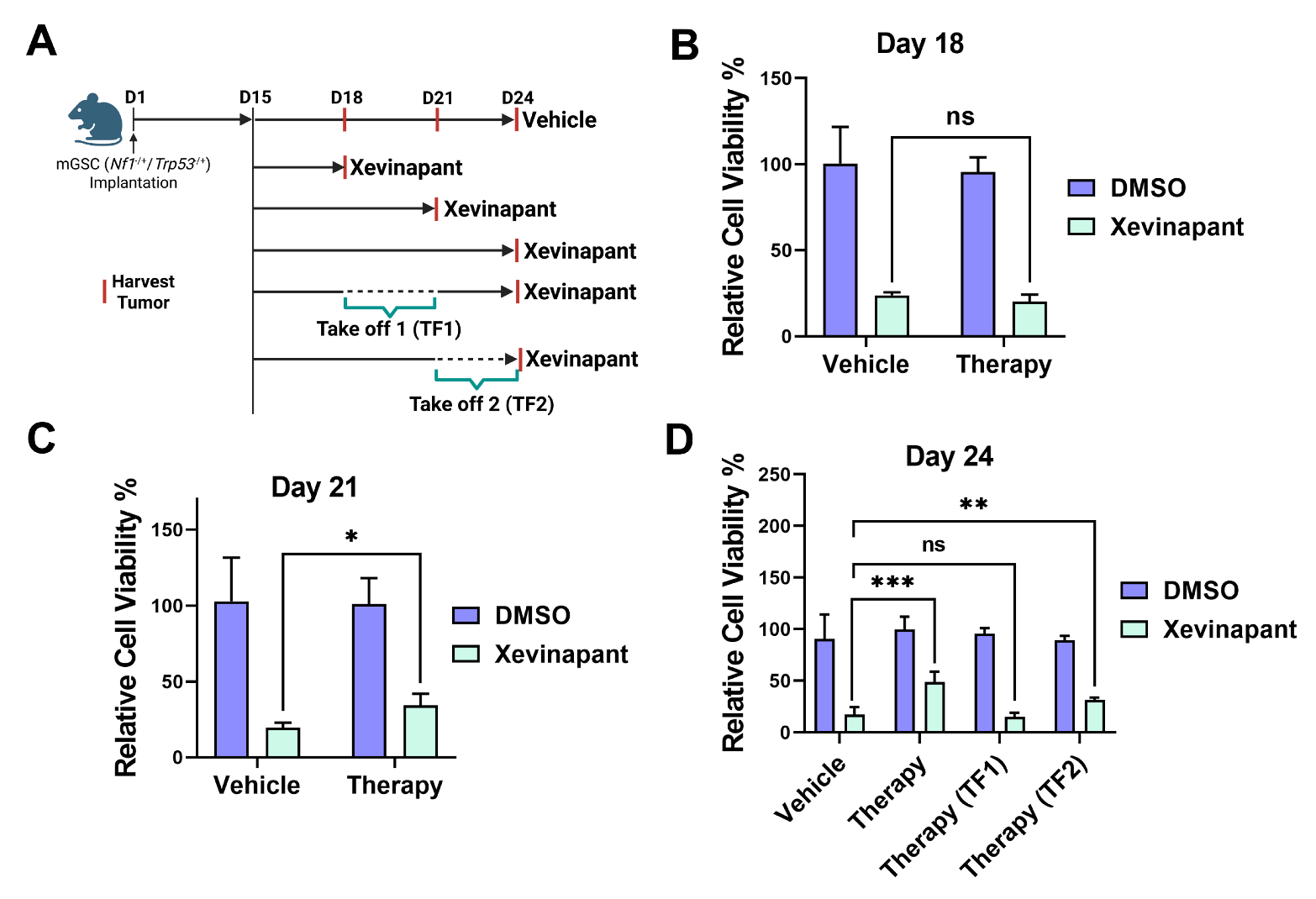


**Fig.S6** A. Timeline of the experiment. B-D. Cells were isolated from animals of vehicle or treatment groups on day 18, day 21 and day 24. Isolated cells were then treated with DMSO or Xevinapant. Relative cell viability was tested after treatment. n=3, Mean ± SEM. *, p<0.05; **, p<0.01; ns, no significance. TF1: Treatment Take off experiment 1; TF2: Treatment Take off experiment 2.

**Supplementary Figure 10**


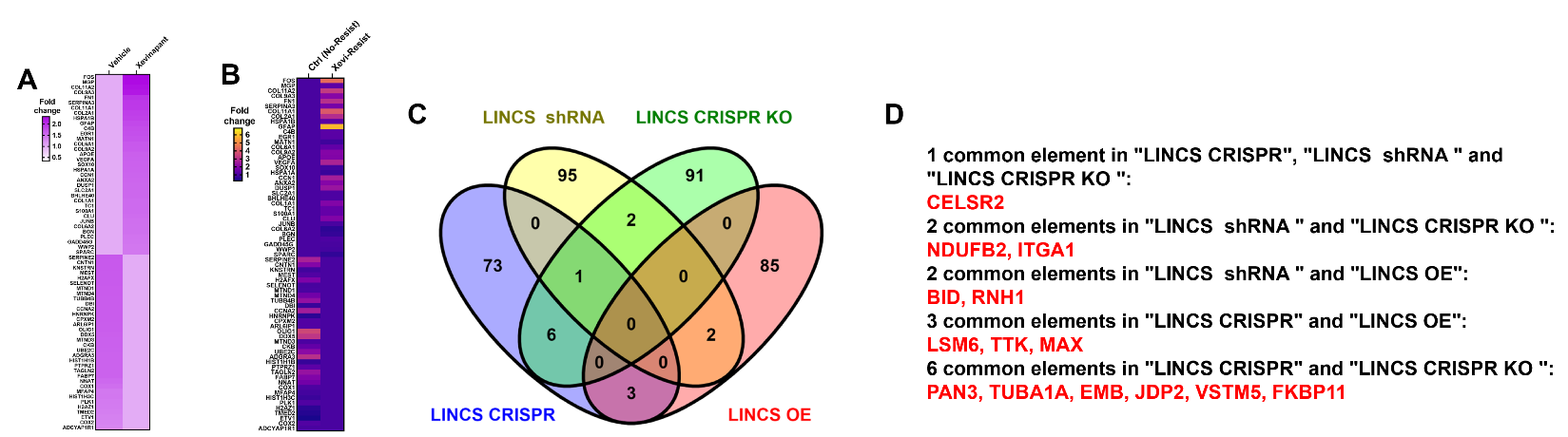


**Fig.S10** A. Heatmap of up/Down regulated gene expression in GBM cells. B. Heatmap of qRT-PCR result to validate the expression of up/down regulated genes in developed Xevinapant-resistant GSCs. C. Venn diagram of cross-analysis of reverser genes from four libraries, LINCS L1000 CRISPR KO Consensus Signatures, LINCS L1000 shRNA Perturbations, LINCS L1000 Overexpression Perturbations and LINCS L1000 CRISPR Perturbations. D. Overlap genes are listed.
