# Supplementary material for: The reprogramming impact of SMAC-mimetic on glioblastoma stem cells and the immune tumor microenvironment evolution
Source: J Exp Clin Cancer Res. 2025 Jul 4;44:191. doi: 10.1186/s13046-025-03452-1 (PMC12231904; doi:10.1186/s13046-025-03452-1)
Supplement: Supplementary file 3 — Supplementary Material 3 [file 13046_2025_3452_MOESM3_ESM.docx]

**Supplemental Methods**

**Single-cell RNA sequencing and analysis**

Single-cell RNA sequencing (scRNA-seq) was performed at Molecular Genomics Core of Moffitt Cancer Center. Sequencing reads were mapped against mm10 mouse transcriptome and processed for UMI counting using Cell Ranger (v3.0, 10X Genomics). Barcodes with UMI counts were imported to Seurat v4.0 for downstream analysis. Cells with less than 200 genes detected, with greater than 10% mitochondrial UMIs, or with complexity score (log10GenesPerUMI) less than 0.8 were filtered out; genes detected in less than three cells were also excluded. Doublets were detected using Scrublet, DoubletFinder, and scDblFinder, using 0.08% doublet rate for every 1,000 cells. Cells identified as doublets by more than one method were removed. Raw UMI counts were log normalized and the top 5,000 variable genes were detected in each sample separately. S and G2/M cell cycle phase scores were assigned to cells using CellCycleScoring function. Individual mice samples were further integrated to remove batch effects using IntegrateData function in Seurat with anchor.features =8,000. Scaled z-scores for each gene were calculated using ScaleData regressing against total reads count, mitochondrial UMIs percentage, cell cycle phases, and log10GenesPerUMI. Principal component analysis was performed on the integrated data and a shared nearest neighbor (SNN) graph was constructed using the first 40 principal components. Clusters were identified using the by Louvain clustering implemented in FindClusters function at resolution=0.8. Uniform manifold approximation and projection (UMAP) was used to visualize gene expression and clusters. Differential expression analysis for each cluster was performed using FindAllMarkers function in Seurat with default settings. Clusters were further annotated by comparing differential genes with canonical markers. Gene signatures for each cluster were listed in Supplemental Table. For marker gene dot plot, gene-level average expression was calculated for each cluster and then Z-score normalized. Differential expression analysis was performed followed by gene set enrichment analysis (GSEA) that compared Xeivinapant vs. Vehicle mice within different cell type/subtype. Genes were ranked based on -log10(p-value)*(sign of log2(fold-change)) resulted from the differential analysis, with most up-regulated genes at the top and most down-regulated genes at the bottom. Pre-ranked GSEA was performed on gene rankings using R package fgsea, against Gene signatures from Hallmark, GO-biological processes and canonical pathways databases from MsigDB.

**Inference of CNV**

To distinguish malignant cells from normal cells, copy number variation was estimated using inferCNV package on <https://github.com/broadinstitute/inferCNV>. Low-expression genes with a median expression below 0.1 were removed. Genes were then annotated according to chromosomal position, and the CNV score was estimated from the moving averages of 100 genes. Lastly, hierarchical clustering was used to distinguish non-malignant cells from malignant cells with clear chromosomal deletions or amplifications.

**Trajectory analysis**

We constructed cell trajectories with Monocle 3 using scRNA-seq data of glioma cells. The dimensionality of the scRNA-seq data was reduced by PCA with 50 components and UMAP was applied to visualize the result of data dimensionality reduction. NPC-like subtype cells were selected as the ‘root’ of the trajectory. Monocle 3 ordered each cell along a learned trajectory according to its transcriptional progress. The default parameters in Monocle 3 were used for the analysis.

**Further analysis of immune cells in scRNA-seq**

We performed scRNA-seq to identify differentially expressed genes between the treatment and non-treatment groups within each cell subgroup. A Wilcoxon rank-sum test was conducted to compare gene expression differences between these two groups. Results were reported in terms of Log2 fold change and adjusted P-values, with significance thresholds set at Log2 fold change ≥ 0.5 and adjusted P-value ≤ 0.05. To rank the differentially expressed genes in each immune cell group, we multiplied each gene's avg_log2FC by -log10(p_val_adj) to generate a composite score. Genes were ranked based on this score, and the top 500 genes with a P-value ≤ 0.05 were selected for further single-cell GO enrichment analysis. GO visualization was performed using the above method. All statistical analyses and visualizations were performed using R (version 4.4.1).

**TME analysis by Flow Cytometry**

Mouse GSC orthotopic model by intracranial injection was established same as scRNA-seq. Mice were euthanized after 7 days treatment (Vehicle and Xevinapant) and tumors were collected for tumor microenvironment (TME) immune profiling by flow cytometry. Single cell suspensions from the harvested tumors were prepared using the Tumor Dissociation Kit, mouse (Miltenyi, CA, USA) and Miltenyi gentleMACS Dissociator following the manufacturer’s protocol. Red blood cells were lysed using RBC Lysis Buffer (BioLegend, CA, USA), and the resulting cells were washed, and resuspended in PBS for flow cytometry analysis. Cell densities within the TME were calculated by (cell number) / (tumor volume). Then, single cell suspensions were first stained with LIVE/DEAD Fixable Dead Cell Stain (Life Technologies, CA, USA) to distinguish live from dead cells. Cells were then washed, pelleted, and resuspended in staining buffer. Cells were preincubated with anti-CD16/CD32 antibody (BD Biosciences, CA, USA) for 15 minutes to prevent nonspecific antibody binding. Subsequently, cells were stained with a combination of fluorescently conjugated anti-mouse monoclonal antibodies for surface phenotyping for 30 minutes at 4°C. Flow cytometry antibodies used in this study were obtained from BD Biosciences and BioLegend. Cell populations were analyzed on a BD LSR II flow cytometer (BD Biosciences) and later using FlowJo software (OR, USA). The following fluorescently conjugated anti-mouse antibodies and their dilutions were used: CD45 (Clone 30-F11, BD Biosciences), CD3 (Clone 17A2, BD Biosciences), CD4 (Clone GK1.5, BD Biosciences), CD8 (Clone 53-5.8, BioLegend), CD11b (Clone M1/70, BD Biosciences), CD11c (Clone N418, BD Biosciences), F4/80 (Clone T45-2342, BD Biosciences), Ly6C (Clone AL-21, BD Biosciences), and Ly6G (Clone 1A8, BD Biosciences).

***Ex vivo* drug response assay**

Mouse GSC orthotopic model by intracranial injection was established. Mice were euthanized after indicated treatment following the timeline in **Fig.S9** and tumors were collected. Single cell suspensions from the harvested tumors were prepared using the Tumor Dissociation Kit, mouse (Miltenyi, CA, USA) and Miltenyi gentleMACS Dissociator following the manufacturer’s protocol. Red blood cells were lysed using RBC Lysis Buffer (BioLegend), and the resulting cells were washed, and resuspended in PBS. The live cells were enriched using Dead Cell Removal Kit (Miltenyi) following the manufacturer’s protocol. Then, plate the cells in 96-well plate at 5,000 cells/well with culture medium. Treatment (DMSO and Xevinapant) was administered the next day and cells were incubated for 48 hours. Following drug treatment, cell viability was assessed using CellTiter-Glo 2.0 Cell Viability Kit (Promega).
